# Unravelling the Role of PIEZO1 in Adipogenesis of Fibrogenic/Adipogenic Progenitors for Muscle Fat Infiltration After Rotator Cuff Tear

**DOI:** 10.1002/jcsm.70004

**Published:** 2025-07-29

**Authors:** Xingzuan Lin, Qingfa Song, Amila Kuati, Zhanghua Li, Hao Zhou, Shujing Feng, Minhui Wang, Siyuan Zhu, Guoqing Cui, Jianhua Wang, Xiexiang Shao

**Affiliations:** ^1^ Department of Orthopaedic Surgery and Spine Center Xinhua Hospital affiliated to Shanghai Jiao Tong University School of Medicine Shanghai China; ^2^ Department of Sports Medicine and Rehabilitation Peking University Third Hospital, Institute of Sports Medicine of Peking University Beijing China; ^3^ Department of Orthopedic Surgery Wuhan Third Hospital, Tongren Hospital of Wuhan University Wuhan China; ^4^ School of Exercise and Health Shanghai University of Sport Shanghai China; ^5^ Department of Bioinformatics, Fujian Key Laboratory of Medical Bioinformatics, School of Medical Technology and Engineering Fujian Medical University Fuzhou China

**Keywords:** adipogenic differentiation, fibrogenic/adipogenic progenitors, low‐intensity pulsed ultrasound, Piezo‐type mechanosensitive ion channel component 1, rotator cuff tears

## Abstract

**Background:**

Muscular fatty infiltration originated from fibrogenic/adipogenic progenitors (FAPs) is a common issue following rotator cuff tear (RCT) that impairs shoulder function. RCT disrupted the biomechanical equilibrium of the shoulder and decreased the mechanical stimuli transmitted to the rotator cuff. Whether mechanical stimuli participate in mediating muscular fatty infiltration after RCT remains unknown. The current study aimed to explore how mechanical environment changes caused by RCT affect muscular fatty infiltration and to identify the potential therapeutic modality.

**Methods:**

Human and murine FAPs were isolated from RCT and control (CTRL) groups to compare adipogenesis properties. Single‐cell RNA sequencing and bulk RNA sequencing were performed to investigate the mechanisms of excessive adipogenesis of FAPs after RCT. The effects and mechanisms of PIEZO1 on adipogenesis of FAPs were investigated by small‐molecule treatment and FAP‐specific PIEZO1 knockout mice. The antiadipogenic effects of low‐intensity pulsed ultrasound (LIPUS) were investigated in vitro and in vivo.

**Results:**

We found that the adipogenic differentiation ability of FAPs was increased after RCT (1.9‐fold vs. CTRL, *p* < 0.05). Single‐cell RNA‐sequencing data analyses and confirmation assays revealed suppressed expression of PIEZO1, which was proved by real‐time qPCR and Western blot (2.9‐fold vs. CTRL, *p* < 0.01, and 3.4‐fold vs. CTRL, *p* < 0.01). There was increased PPARG expression and adipogenesis ability of FAPs after PIEZO1 ablation (1.7‐fold vs. CTRL, *p* < 0.01, and 2.3‐fold vs. CTRL, *p* < 0.01). The PIEZO1‐mediated antiadipogenic role by ERK/KLF4 signalling was confirmed by small‐molecule treatment and *PIEZO1 KO* mice evaluation. LIPUS could reactivate PIEZO1 and mitigate the adipogenesis of FAPs in vitro, ameliorate the muscular fatty degeneration after RCT (3.0‐fold vs. CTRL, *p* < 0.001) and facilitate the improvement of shoulder functions.

**Conclusions:**

Our findings indicate that the downregulation of PIEZO1 expression contributes to the enhanced adipogenesis capacity of FAPs after RCT by inhibiting ERK/KLF4 signalling. LIPUS could mitigate the excessive adipogenesis of FAPs by upregulating expression of PIEZO1, alleviate muscular fatty infiltration and improve shoulder function after RCT.

## 1 Introduction

Rotator cuff tear (RCT) is a prevalent musculoskeletal disease associated with pain and disability of the shoulder, affecting approximately 22.1% of the general population [1]. Although surgical repair constitutes a primary treatment for massive RCT, its effectiveness remains unsatisfactory. Studies have reported that the retearing rate after RCT repair was up to 94%, which can be partly attributed to the severe muscular fatty degeneration [2, 3]. Fatty infiltration significantly exacerbates muscular function in RCT patients [4], diminishes the efficacy of surgery and contributes to the progression towards irreversible RCT [5]. Thus, clarifying the intricate mechanism of muscular fatty infiltration after RCT would greatly facilitate the development of treatment strategies for RCT.

Fibrogenic/adipogenic progenitors (FAPs), which play pivotal roles in muscular regeneration and homeostasis, notably express the platelet‐derived growth factor receptor alpha (PDGFRα) and exhibit multilineage potential including adipogenesis and fibrogenesis [6]. FAPs have been identified as major contributors to the development of ectopic muscular fatty infiltration under pathological conditions [7], such as RCT [8], Duchenne muscular dystrophy [6] and Type II diabetes mellitus [9]. Therefore, it is of paramount interest to impede the adipogenesis of FAPs and mitigate muscular fatty infiltration after RCT. However, the molecular mechanism underlying the adipogenesis of FAPs after RCT remains incomplete.

RCT is characterized by painful limitations in range of motion and poses a significant clinical challenge in orthopaedic surgery [10]. A previous study validated that RCT disrupted the biomechanical equilibrium of the shoulder, subsequently reducing the mechanical stimuli transmitted to skeletal muscles through the tendon [11]. Mechanical force is required to maintain the muscular quality [12]. Nevertheless, whether mechanical stimuli participate in mediating FAP adipogenesis after RCT remains unknown.

Here, we unveiled an elevated adipogenic potential and suppressed expression of PIEZO1 in FAPs after RCT. The decreased expression of PIEZO1 could contribute to the adipogenesis of FAPs via inhibiting ERK/KLF4 pathways. Moreover, low‐intensity pulsed ultrasound (LIPUS) could activate PIEZO1 and ameliorate muscular fatty infiltration after RCT. This study underscores that PIEZO1 is involved in FAP adipogenesis, and LIPUS could serve as a non‐invasive and non‐surgical treatment approach for mitigating muscular fatty infiltration after RCT.

## 2 Methods and Materials

### 2.1 Animals

All experimental procedures involving animals were performed in compliance with the approval of the Animal Ethics and Welfare Committee of the local institution (Approval No. XHEC‐F‐2023‐027). Forty wild‐type male mice (C57BL/6, 12‐week‐old) and *Piezo1 f/f* mice were purchased from GemPharmatech (Nanjing, China). PDGFRα–CreERT2 mice were procured from The Jackson Laboratory. Conditional gene knockout mice were induced in the third week with a daily dose of 200 μL of 10‐mg/mL tamoxifen (ABCONE, Cat. No. T56488) for 1 week.

The RCT animal models were conducted as previously described [13]. Briefly, the tendons of the supraspinatus and infraspinatus muscles in the right shoulder were entirely transected near the humeral head. In the left shoulder, a sham surgery was conducted without tendon detachment to serve as a control. For shoulder function assays, both shoulders were injured to establish RCT models. In the rescue experiment, LIPUS (1 MHz burst wave) was exerted every 3 days for 6 weeks.

### 2.2 Human Samples

The procedure was approved by the ethics committee in our institution (Approval No. XHEC‐D‐2022‐129). Muscle samples with or without RCT were collected from patients who underwent arthroscopic repair, total reverse shoulder replacement or tendon transfer surgery [14]. The sample collection procedure did not result in any additional complications for the patients. The informed consent was obtained and signed by all participants.

### 2.3 Isolation of FAPs

The digestion procedure for skeletal muscle was performed as previously reported [15]. Briefly, fresh muscles were mechanically minced and digested with collagenase II (Worthington Biochemical, 700–800 U/mL, Cat. No. LS004177) for 1 h. Subsequently, the muscle fragments were treated with a mixture of collagenase II (70–80 U/mL) and Dispase (Life Technologies, Cat. No. 17105041, 11 U/mL) for 30 min. The single‐cell suspension was then filtered through a 70‐ and 40‐μm strainer (BD Falcon). Red blood cells were removed using a red cell lysis buffer (Thermo Fisher Scientific, Cat. No. 00‐433‐57). For the isolation of mouse FAPs, the single‐cell suspensions were stained by APC anti‐mouse CD45 (BioLegend, Cat. No. 103112), APC anti‐mouse CD31 (BioLegend, Cat. No. 102510), FITC‐anti‐mouse LY‐6A/E (SCA‐1) and PE anti‐mouse PDGFRα (Thermo Fisher Scientific, Cat. No. 12‐1401‐81) for 45 min at 4°C with shaking. The subset of CD45^−^CD31^−^SCA‐1^+^PDGFRα^+^ murine FAPs was isolated with a BD Influx sorter (BD Biosciences) [15]. For the isolation of human FAPs, the single‐cell suspension was stained with PerCP/Cy5.5 anti‐human CD31 (BioLegend, Cat. No. 30313), PE‐Cy5 anti‐human CD45 (BD Biosciences, Cat. No. 555484), BV421 anti‐human CD56 (BioLegend, Cat. No. 562751) and APC anti‐human CD34 (BioLegend, Cat. No. 343510) for 45 min under shaking. Finally, the subgroup of CD31^−^CD45^−^CD56^−^CD34^+^ cells was obtained by a BD Influx sorter [9].

### 2.4 Cell Culture, Treatment and Differentiation

Primary human and mouse FAPs were cultured in Matrigel‐coated (Biocoat, Cat. No. 354277) plates with α‐MEM (Corning, Cat. No. 10‐022‐CV) containing 10% FBS (Gibco, Cat. No. 10‐013‐CV), 2.5‐ng/mL bFGF (R&D, Cat. No. 233‐FB‐025) and 1% penicillin–streptomycin (Gibco, Cat. No. 15140‐122) at 37°C with 5% CO_2_; 2.5‐μM Yoda1 (MedChemExpress, Cat. No. HY‐18723), 2.5‐μM GsMTx4 (MedChemExpress, Cat. No. HY‐P1410), 5‐μM TBHQ (MedChemExpress, Cat. No. HY‐100489) and 1‐μM U0126 (MedChemExpress, Cat. No. HY‐12031) were applied to investigate the physiological role of PIEZO1 in FAP adipogenesis and related mechanisms. For the adipogenic induction experiment, FAPs were treated with an adipogenic induction medium (AIM) composed of high‐glucose DMEM (Gibco, Cat. No. 12100‐046) supplemented with 20% FBS, 0.5‐mM 3‐isobutyl‐1‐methylxanthine (Sigma‐Aldrich, Cat. No. I5879), 0.25‐μM dexamethasone (Sigma‐Aldrich, Cat. No. D4902), 1‐μg/mL insulin (Sigma‐Aldrich, Cat. No. I2643) and 1% penicillin–streptomycin. In the LIPUS treatment experiment, LIPUS (1 MHz, 1‐Hz pulse repetition frequency) was applied for 5 min at a time, every day.

### 2.5 Single‐Cell RNA Sequencing

Single‐cell suspension was first isolated from discarded rotator muscles in patients with or without RCT, with two samples included from each group (Table S1). The single‐cell RNA‐sequencing procedure and analysis were conducted as previously described [16]. Live cells were stained with propidium iodide (Sangon Biotech, Cat. No. E607328) and Hoechst (Sangon Biotech, Cat. No. A601112) and subsequently sorted by FACS. Chromium Single Cell 3′ Reagent Kits (10x Genomics, Cat. No. 1000121‐1000157) were prepared for library construction, and sequencing was performed on the Illumina NovaSeq 6000 platform (Illumina). Data analysis was carried out on a bioinformatics analysis platform (Majorbio, China). Initially, cells that had fewer than 200 genes, over 7500 genes, or exhibited more than 10% mitochondrial gene content were excluded from subsequent analyses. Following rigorous quality control measures, a total of 33 033 cells were retained for further evaluation.

To perform cell clustering, the ‘FindClusters’ function was employed with a resolution setting of 0.05. Dimensionality reduction was achieved using the ‘RunUMAP’ function. Ultimately, the analysis yielded six distinct clusters of cells. To discern cell types, the clustering results were further analysed with the ‘FindClusters’ function. Marker genes for each identified cluster were determined through the ‘FindAllMarkers’ function, with the criteria being *p* values < 0.05 and |logFC| > 0.25 to classify them as significant marker genes. The identification of cell types was based on the expression profiles of established canonical marker genes.

Differential gene expression was assessed using the ‘FindMarkers’ function to compare the gene profiles of patients in the RCT and control (CTRL) groups, employing the Wilcoxon test for statistical analysis. Genes were considered significantly differentially expressed if they met the criteria of *p* value < 0.05 and |logFC| > 0.25. Pathway enrichment analysis for the differentially expressed genes (DEGs) was conducted using the clusterProfiler package [17, 18]. Gene Ontology (GO) enrichment analysis was performed utilizing goatools, which can be accessed at https://github.com/tanghaibao/goatools.

### 2.6 Bulk RNA Sequencing and Analysis

RNA‐sequencing libraries were prepared using the NEBNext Ultra RNA Library Prep Kit for Illumina (NEB, Cat. No. E7530L). The paired‐end libraries were sequenced on an Illumina NovaSeq 6000 platform, utilizing a read length of 2 × 150 bp. Raw paired‐end reads underwent quality enhancement and control using fastp with default settings. Genes were deemed significantly differentially expressed if they met the criteria of *p* value < 0.05 and |logFC| > 1, as assessed by DEGseq. Additionally, GO and Kyoto Encyclopedia of Genes and Genomes (KEGG) analyses were performed using goatools and KOBAS [19, 20].

### 2.7 Immunofluorescent Staining

The FAPs and embedded muscles were transversally sectioned into 10‐μm‐thick slices by Leica cryostat. Immunofluorescence staining was performed according to established procedures. Muscle tissue and cell samples were fixed in 4% paraformaldehyde (PFA) for 20 min, permeabilized with 0.5% Triton X‐100 for 30 min at room temperature under shaking and blocked by 1% BSA (Beyotime Biotechnology, Cat. No. ST023) for 1 h. Primary anti‐PDGFRα (Abcam, Cat. No. ab203491), anti‐PPARγ (Cell Signaling Technology, Cat. No. 2435), anti‐PDGFRα (R&D Systems, Cat. No. AF1062), anti‐Laminin α‐2 (Santa Cruz Biotechnology, Cat. No. sc‐59854) and anti‐perilipin A/B (Millipore, Cat. No. P1873) were applied to stain overnight at 4°C. Secondary antibodies, Alexa Fluor goat anti‐rat 647 (Acmec, Cat. No. AC52050) and Alexa Fluor goat anti‐rabbit and anti‐mouse 594 or 488 (Invitrogen, 1:500), were incubated at room temperature for 1 h. 4′,6‐Diamidino‐2‐phenylindole (DAPI, Vector Laboratories, Cat. No. H‐1200) was applied for staining the nucleus.

### 2.8 Oil Red O (ORO) Staining

The ORO solution (1 mg/mL dissolved in isopropanol) was first filtered by a 0.45‐μm strainer. Cultured cells were fixed with 4% PFA for 20 min and permeabilized with 0.5% Triton X‐100 for 30 min. Subsequently, the nucleus was counterstained with DAPI for 5 min. Finally, the cells were stained with ORO solution for 15 min at room temperature under shaking. All the fluorescent images were acquired using an IX73 fluorescence microscope and were analysed with ImageJ or Image‐Pro Plus 6.0 software in a blind manner. To quantify the lipid content, isopropanol was used to extract the ORO from the stained cells. The absorbance at 496 nm was measured by a microplate reader (Thermo Fisher Scientific).

### 2.9 Overexpression, RNAi and Dual‐Luciferase Reporter Assay

A total of 2 μL of Lipofectamine 2000 (Invitrogen, Cat. No. 11668019) was combined with 200 μL of Opti‐MEM I Reduced Serum Medium (Thermo Fisher, Cat. No. 31985062) along with 1‐μg KLF4 and PIEZO1 plasmids. This mixture was allowed to incubate at room temperature for 20 min. Following this, it was evenly distributed into a 12‐well plate culturing FAP cells. Transfection protocols for siRNA were similar to those for overexpression of plasmid. The siRNA sequence was as follows: KLF4: 5′‐AGAUCGUUGAACUCCUCGG‐3′; PIEZO1: 5′‐ACAUAGAUCCAGUACUUGG‐3′.

For the dual‐luciferase reporter assay, cells were transfected with a luciferase reporter plasmid (Tsingke Technology) containing the promoter region of the target gene, along with either a KLF4 overexpression plasmid or KLF4‐specific mutant plasmid. The predicted site 5′‐CCCCACCC‐3′ was firstly mutated to 5′‐AAGTAAGT‐3′. A Renilla luciferase plasmid was cotransfected as an internal control to normalize transfection efficiency. After 48 h, luciferase activity was measured using a dual‐luciferase reporter assay system, according to the manufacturer's protocol. The firefly luciferase activity was normalized to Renilla luciferase activity, and relative luciferase activity was compared between the overexpression and CTRL groups to assess the effect of KLF4 on the target gene promoter.

### 2.10 Chromatin Immunoprecipitation (ChIP)–qPCR Assay

ChIP was performed using the SimpleChIP Plus Kit (CST, Cat. No. 9005S). Briefly, FAP cells were fixed with 1% formaldehyde for 10 min, followed by lysis and sonication. For the immunoprecipitation step, antibodies targeting KLF4 (Proteintech, Cat. No. 11880‐1‐AP) or rabbit IgG (CST, Cat. No. 2729) were utilized. The obtained DNA fragments after immunoprecipitation were subsequently quantified using ChIP–qPCR. The primer sequences were as follows: forward primer: 5′‐GACAGGTCACGATGGACAGC‐3′; reverse primer: 5′‐TTAAATTTCTTAGCAGTGTT‐3′.

### 2.11 Triglyceride Assay

The Triglyceride Colorimetric Assay Kit (Elabscience, Cat. No. E‐bc‐K261‐M) was used to assess the lipid content after tendon injury. The muscle tissue was first grounded with isopropanol (Acmec, Cat. No. I86620) to extract the lipids. Then, tissue fragments were separated by centrifugation at 10 000 *g* for 3 min, and the supernatant was used for measuring the triglycerides at the absorbance of 510 nm.

### 2.12 Real‐Time qPCR

Total RNA was isolated by the EZ‐press RNA Purification Kit (EZBioscience, Cat. No. B0004DP) as per the manufacturer's instructions. One microgram of RNA was reverse transcribed into cDNA using MuLV reverse transcriptase (NEB, Cat. No. M0253L). The cDNA products were applied as templates of real‐time qPCR using Genious 2X SYBR Green Fast qPCR Mix (ABclonal, Cat. No. RK21205) in the CFX96 Real‐Time PCR System. The primers are listed as follows: mouse‐Piezo1‐F: 5′‐CCTGTTACGCTTCAATGCTCT‐3′; mouse‐Piezo1‐R:5′‐GTGTAGGCATATCTGAAAGGCAA‐3′; mouse‐Plin1‐F: 5′‐CTGTGTGCAATGCCTATGAGA‐3′; mouse‐Plin1‐R: 5′‐CTGGAGGGTATTGAAGAGCCG‐3′; mouse‐Cebpa‐F: 5′‐GCGGGAACGCAACAACATC‐3′; mouse‐Cebpa‐R: 5′‐GTCACTGGTCAACTCCAGCAC‐3′; mouse‐Fabp4‐F: 5′‐AAGGTGAAGAGCATCATAACCCT‐3′; mouse‐Fabp4‐R: 5′‐TCACGCCTTTCATAACACATTCC‐3′; mouse‐Pparg‐F: 5′‐GGAAGACCACTCGCATTCCTT‐3′; mouse‐Pparg‐R: 5′‐GTAATCAGCAACCATTGGGTCA‐3′; mouse‐Klf4‐F:5′‐GGCGAGTCTGACATGGCTG‐3′; mouse‐Klf4‐R: 5′‐GCTGGACGCAGTGTCTTCTC‐3′; mouse‐Gapdh‐F: 5′‐AGGTCGGTGTGAACGGATTTG‐3′; mouse‐Gapdh‐R: 5′‐GGGGTCGTTGATGGCAACA‐3′; human‐PIEZO1‐F: 5′‐CAGGCCTATGAGGAGCTGTC‐3′; human‐PIEZO1‐R: 5′‐TTGTAGAGCTCCCGCTTCAT‐3′; human‐GAPDH‐F: 5′‐CAAGGCTGAGAACGGGAAGC‐3′; human‐GAPDH‐R: 5′‐AGGGGGCAGAGATGATGACC‐3′; human‐KLF4‐F: 5′‐CAGCTTCACCTATCCGATCCG‐3′; and human‐KLF4‐R: 5′‐GACTCCCTGCCATAGAGGAGG‐3′.

### 2.13 Western Blot

Total proteins were extracted by Western and IP lysis (Beyotime, Cat. No. P0013). They were then subjected to electrophoretic separation on polyacrylamide gels and transferred to the nitrocellulose membranes. After 1 h of blocking with 5% BSA, the primary antibodies were incubated overnight at 4°C. Primary antibodies used in this study included anti‐PPARγ (Cell Signaling Technology, Cat. No. 2435), anti‐FABP4 (Proteintech, Cat. No. 12802‐1‐AP), anti‐GAPDH (Cell Signaling Technology, Cat. No. 5174), anti‐ERK1/2 (Cell Signaling Technology, Cat. No. 4695T), anti‐p‐ERK1/2 (Cell Signaling Technology, 4370T), anti‐KLF4 (Proteintech, Cat. No. 11880‐1‐AP) and anti‐PIEZO1 (Proteintech, Cat. No. 28511‐1‐AP). After washing the membranes three times with TBST 1×, an HRP‐conjugated secondary anti‐rabbit and mouse IgG (Proteintech, Cat. No. RGAR001 and RGAM001) was incubated for 1 h. The LumiQ ECL liquid (ShareBio, Cat. No. SBWB012) was used to visualize specific bands by Gel Doc XR (Bio‐Rad).

### 2.14 Gait Analysis

The gait analysis of mice with RCT was performed using the Noldus CatWalk system as previously described [21]. The starting point of the walkway was inclined at a 10° angle in relation to the horizontal plane, enabling the mouse to navigate the entire course at their discretion. Before the formal experiment, the mice underwent tunnel navigation training at no less than 10 m/s. A digital camera positioned below the walkway automatically captured and documented images of the paw prints. This experiment recorded various gait parameters, including the stride length, stance width and paw area at the peak stance. The stride length indicates the abduction of the shoulder of mice, the stance width reflects the limb load‐bearing capacity, and the paw area at peak stance represents chronic pain [22]. The functional consequences of shoulder in RCT mice could be assessed by analysing these parameters.

### 2.15 Treadmill Analysis

The treadmill apparatus (Zll‐PT/5 S) was employed for treadmill analysis as previously described [15]. The mice were first familiarized with the treadmill apparatus before the formal experiments. For the endurance experiment, the mice ran on the treadmill at a 20‐m/min speed. In exhaustion experiments, the speed was set at 16 m/min in the beginning and increased at a rate of 2 m/min every 3 min. The termination of both tests was recognized when the mice stopped running for 10 s even with electrical stimulus.

### 2.16 Statistical Analysis

The reliability and reproducibility of the results were introduced in figure legends. To minimize the bias, the operator remained blind to the group information during data analysis and evaluation. The randomization was performed by SPSS software. All experimental data were revealed by mean ± SD. Student's *t* test or one‐way ANOVA in GraphPad Prism 8 software was performed to verify the difference between groups. The threshold of statistical difference was set as *p* < 0.05.

## 3 Results

### 3.1 There Was Decreased Expression of PIEZO1 and Increased Adipogenesis Potential of FAPs After RCT

**FIGURE 1:**
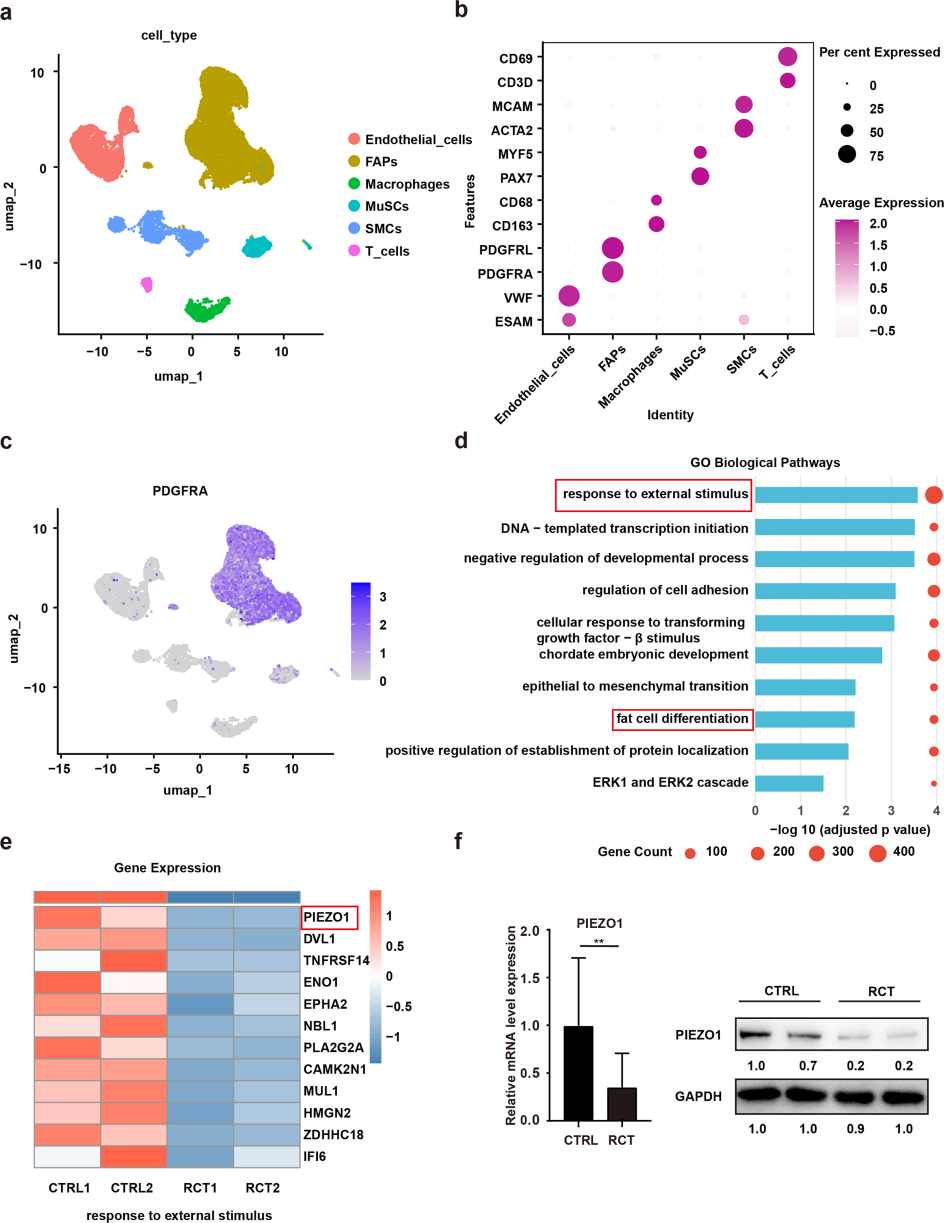
The scRNA‐seq indicated increased adipogenesis and reduced mechanical stimulus of fibrogenic/adipogenic progenitors (FAPs) after RCT. (a) The UMAP plots of cell clusters. Muscle tissues harvested from patients with or without RCT were used for cell collection and subsequent scRNA‐seq. A total of 33 033 cells were classified into six groups. (b) Dot plot of markers used for classification. The dot sizes revealed the percentage of specific gene expressions in various cell types. The dot colours indicated the gene expression levels. (c) UMAP plots of marker gene PDGFRA in FAPs. (d) Bar diagram of GO enrichment analysis of upregulated and downregulated genes for FAPs after RCT. (e) Heat map illustrating differential expression of genes within the ‘response to external stimulus’ GO term in FAPs from RCT and CTRL. (f) Relative mRNA and protein expression of PIEZO1 in human FAPs with or without RCT. RT‐qPCR: CTRL, *n* = 8; RCT, *n* = 17. Western blot: *n* = 3. CTRL, control group; MuSCs, muscle stem/progenitor cells; SMCs, smooth muscle cells. **p < 0.001.

Accumulated studies indicated the occurrence of ectopic fatty degeneration in rotator cuff muscles after RCT [8, 23]. To investigate the mechanism of muscular fatty infiltration after RCT, we first procured discarded muscles from individuals with or without RCT for single‐cell RNA sequencing. In the scRNA‐sequencing analysis, cells were categorized into six clusters (Figure 1a,b). Because FAPs play a crucial role in fatty infiltration after RCT, we mainly focused on the FAPs that highly expressed PDGFRα (Figure 1b,c). Further GO enrichment analysis of DEGs in FAPs revealed enrichment in response to external stimuli and fat cell differentiation (Figure 1d). To validate these GO analysis results, we employed a murine RCT model to isolate FAPs from mice with and without RCT via FACS (Figure S1). Fresh mice FAPs isolated from the RCT and CTRL group were subjected to induction with an adipogenic cocktail. In harmony with the results of single‐cell RNA‐sequencing analysis, the ORO staining and quantification assay results revealed a notably greater ORO‐positive area in the RCT group (Figure S2a–c). In addition, gene expressions of adipogenic differentiation markers Plin1, Cebpa and Pparg were substantially elevated in the RCT group (Figure S2d). Consistently, the protein level of adipogenic‐related markers PPARG and FABP4 was also increased (Figure S2e). These findings unveiled an increased adipogenic differentiation ability in FAPs isolated from the RCT group compared to those from the CTRL group.

To elucidate the mechanisms underlying this enhanced adipogenic differentiation, we further investigated DEGs within the ‘response to external stimulus’ GO term and in the scRNA‐sequencing data comparing CTRL and RCT groups. Notably, we observed a decrease in mechanosensitive ion channel 1 (PIEZO1) expression in FAPs after RCT (Figure 1e). Consistently, the RT‐qPCR and Western blot results also found a significantly suppressed expression of PIEZO1 in human FAPs after RCT (Figure 1f). Because PIEZO1 has been implicated in inhibiting adipogenesis of bone mesenchymal stem cells [24], the decreased expression of PIEZO1 in FAPs after RCT might be closely associated with its excessive adipogenesis. Taken together, these data confirmed that there was a decreased expression of PIEZO1 and increased adipogenic differentiation potential in FAPs after RCT.

### 3.2 The PIEZO1 Is Required for Regulating Adipogenesis of FAPs

**FIGURE 2:**
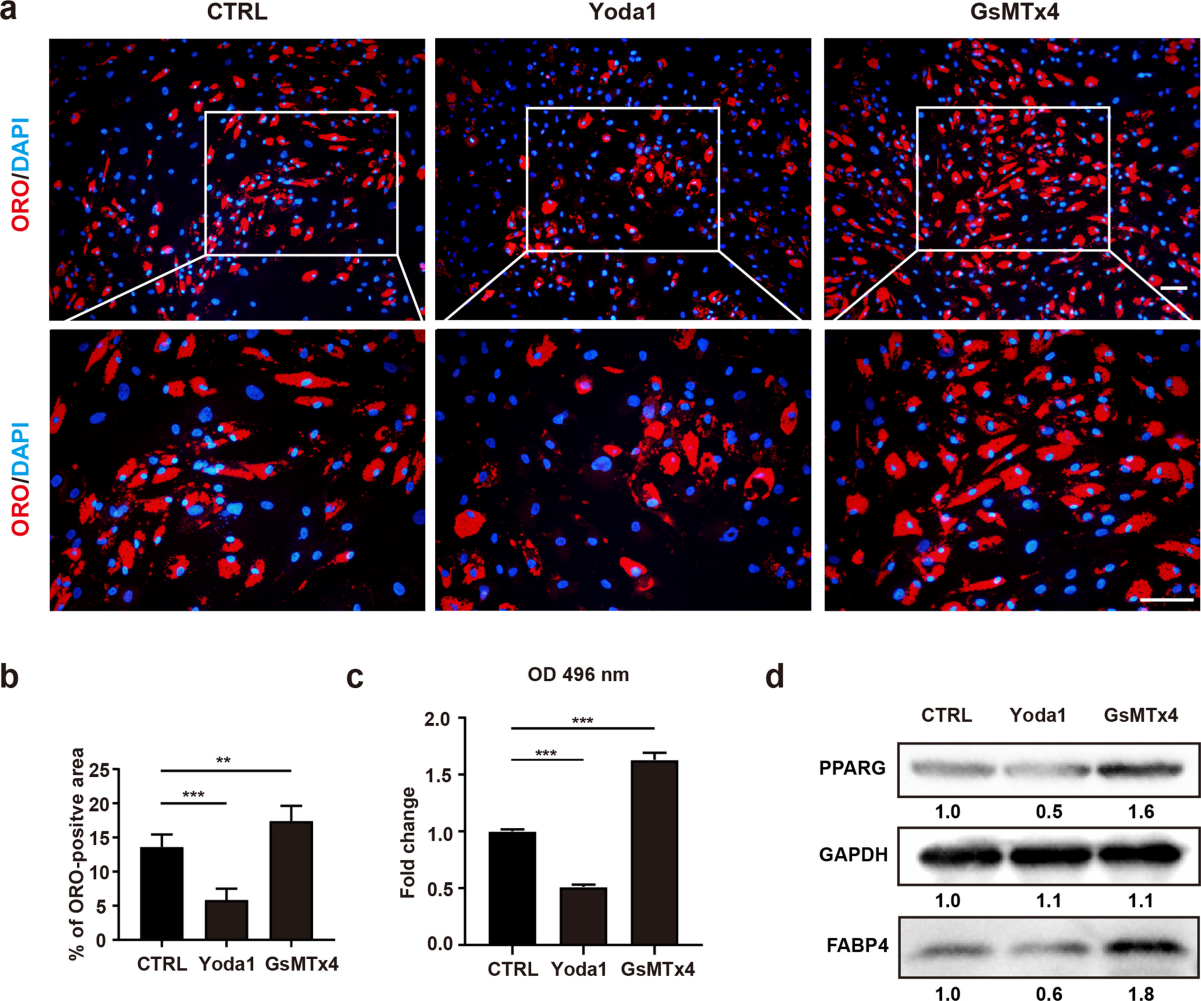
The PIEZO1 is required for the adipogenesis of FAPs. (a) The Oil Red O (ORO) staining of human FAPs after adipogenic differentiation with adipogenic induction medium (AIM) for 10 days (*n* = 6). Freshly isolated human FAPs were cultured in AIM (CTRL), AIM + Yoda1 (PIEZO1 agonist; Yoda1) and AIM + GsMTx4 (Piezo1 inhibitor; GsMTx4). Scale bar = 100 μm. (b) Quantitative analysis of ORO‐positive area of human FAPs cultured in AIM (CTRL), AIM + Yoda1 (Yoda1) and AIM + GsMTx4 (GsMTx4) (*n* = 6). (c) Absorbance measurement of differentiated FAPs with different treatments after ORO staining (*n* = 3). Human FAPs were grown in AIM, AIM + Yoda1 and AIM + GsMTx4 for 10 days. (d) The relative protein expression of PPARG, GAPDH and FABP4 of FAPs treated with CTRL, Yoda1 and GsMTx4 (*n* = 3). **p < 0.01 and ***p < 0.001.

To explore the role of PIEZO1 in FAP adipogenesis, the healthy human FAPs were first induced with an adipogenic cocktail, along with the addition of PIEZO1 agonist Yoda1 and inhibitor GsMTx4. ORO staining assays revealed that PIEZO1 agonist Yoda1 inhibited adipogenic differentiation, whereas inhibition of PIEZO1 increased the adipogenic potential of human FAPs (Figure 2a–c). Consistently, the expressions of adipogenic proteins, including PPARG and FABP4, were inhibited by the PIEZO1 agonist Yoda1 and increased by the PIEZO1 inhibitor GsMTx4 (Figure 2d).

To corroborate the effects of PIEZO1 on FAP adipogenesis, we conducted PIEZO1 knockdown and overexpression experiments in human FAPs. In agreement with the agonist/inhibitor data, PIEZO1 knockdown demonstrably enhanced adipogenic differentiation, as evidenced by increased lipid droplets by ORO staining (Figure S3a–c). Conversely, PIEZO1 overexpression notably diminished adipogenic differentiation, as indicated by decreased lipid droplets by ORO staining (Figure S3a–c). Moreover, PIEZO1 knockdown resulted in elevated expression of the adipogenic proteins PPARG and FABP4 (Figure S3d). These findings collectively reinforce the conclusion that PIEZO1 exerts a negative regulatory influence on FAP adipogenesis.

**FIGURE 3:**
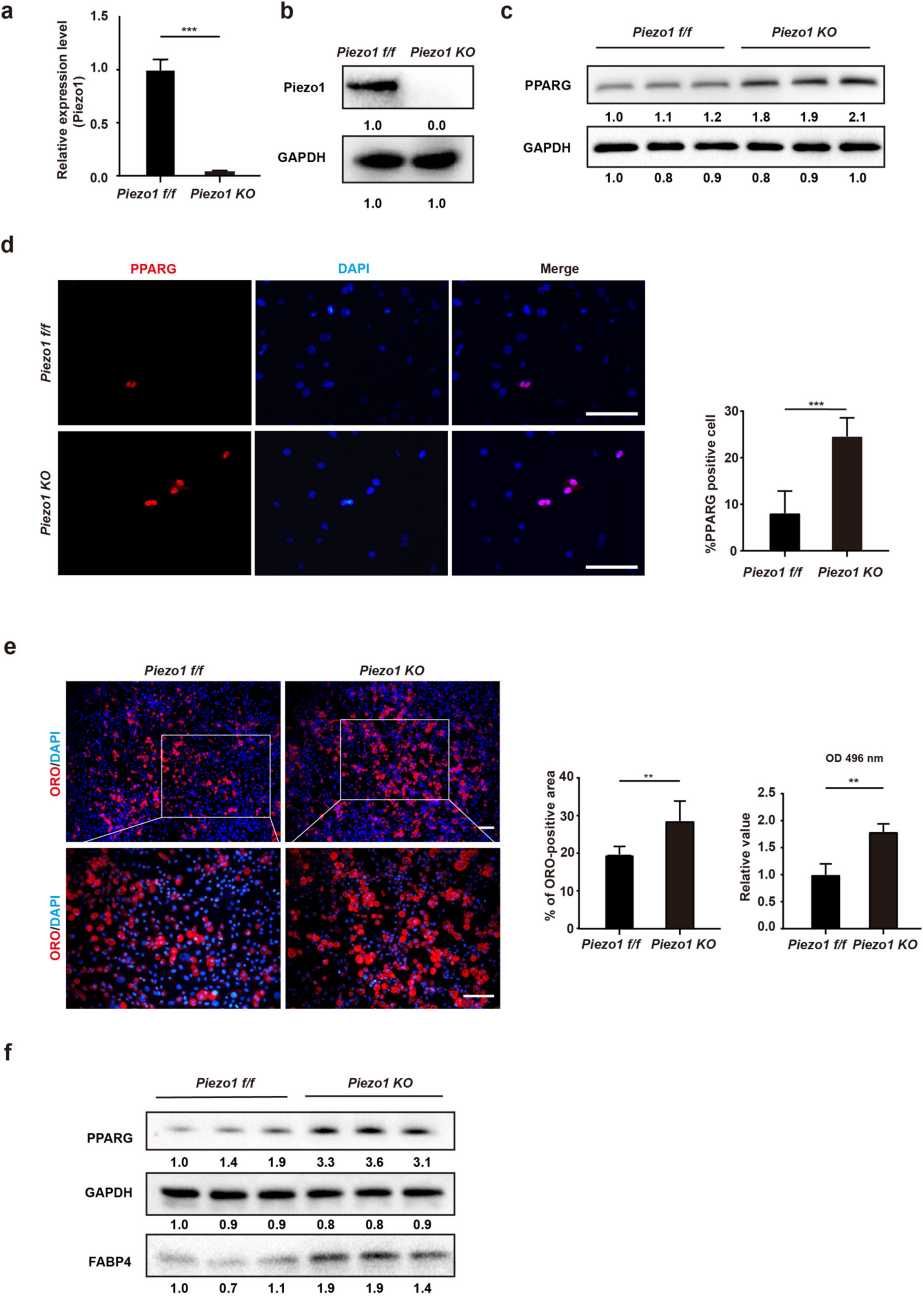
The ablation of PIEZO1 increased the expression of PPARG and promoted the adipogenesis of FAPs. (a) The mRNA expression of Piezo1 in FAPs isolated from *Piezo1 f/f* and *Piezo1 KO* mice was measured by RT‐qPCR analysis (*n* = 3). (b) The protein expression of Piezo1 and GAPDH in *Piezo1 f/f* and *Piezo1 KO* mice (*n* = 3). (c) The protein expression of PPARG and GAPDH in FAPs isolated from *Piezo1 f/f* and *Piezo1 KO* mice (*n* = 3). (d) The immunofluorescence staining of PPARG in freshly isolated FAPs from *Piezo1 f/f* and *Piezo1 KO* mice (*n* = 5). Scale bar = 100 μm. (e) Oil Red O (ORO) staining and statistical analysis of FAPs after 7‐day adipogenic induction (*n* = 6). FAPs were isolated from *Piezo1 f/f* and *Piezo1 KO* mice. Scale bar = 100 μm. Absorbance measurement of differentiated FAPs from *Piezo1 f/f* and *Piezo1 KO* groups after ORO staining (*n* = 3). (f) The relative protein expression of PPARG, GAPDH and FABP4 in FAPs from *Piezo1 f/f* and *Piezo1 KO* mice treated with adipogenic induction (*n* = 3). **p < 0.01 and ***p < 0.001.

To further confirm the role of PIEZO1 in FAP adipogenesis after RCT, FAP‐specific Piezo1 conditional knockout mice (*Piezo1 KO*) were generated by crossing PDGFRα–CreERT2 and *Piezo1 f/f* mice. After tamoxifen induction, *Piezo1 KO* FAPs were isolated, and the efficiency of Piezo1 knockout was confirmed by RT‐qPCR and Western blot (Figure 3a,b). There was an elevated number of Pparg‐positive FAPs observed in supraspinatus muscles after Piezo1 knockdown (Figure S4a,b). We also discovered that the protein expression of PPARG was increased after Piezo1 knockout (Figure 3c). Immunofluorescent staining further demonstrated that *Piezo1 KO* FAPs exhibited an increased proportion of Pparg‐positive cells in vitro (Figure 3d). These data indicated enhanced adipogenesis potential of FAPs after Piezo1 knockout. Subsequently, the *Piezo1 KO* FAPs were cultured with an adipogenic cocktail for 7 days. ORO assays revealed that there existed more lipids after adipogenic differentiation of *Piezo1 KO* FAPs (Figure 3e). Western blot results also showed that the adipogenic proteins (PPARG and FABP4) were upregulated in the Piezo1 ablation group (Figure 3f).

Combined, these results suggest that Piezo1 is involved in adipogenesis of FAPs, and the decreased Piezo1 enhances the adipogenic differentiation potential of FAPs.

### 3.3 Decreased Piezo1 Contributes to Adipogenesis of FAPs by Mediating MAPK/ERK Pathways

**FIGURE 4:**
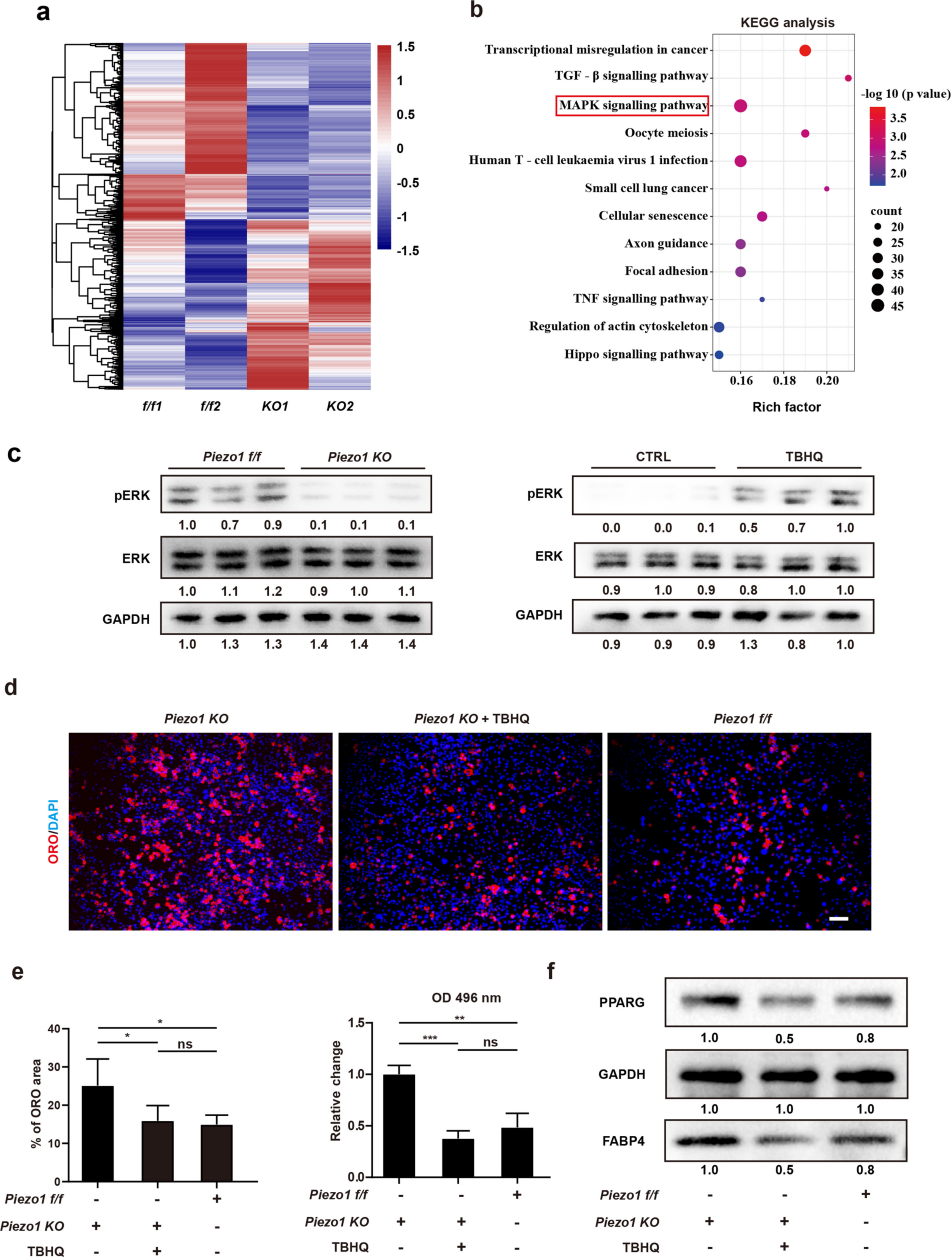
Decreased Piezo1 contributes to adipogenesis of FAPs by mediating MAPK/ERK pathways. (a) Heat map of differentially expressed genes in FAPs obtained from *Piezo1 KO* and *Piezo1 f/f* mice. (b) Bubble chart of KEGG analysis for downregulated genes of murine FAPs from *Piezo1 KO* mice when compared with those in *Piezo1 f/f* FAPs. (c) The protein expression of ERK1/2, p‐ERK1/2 and GAPDH in FAPs isolated from *Piezo1 KO* and *Piezo1 f/f* mice. The protein expression of ERK1/2, p‐ERK1/2 and GAPDH in *Piezo1 f/f* FAPs, *Piezo1 KO* FAPs and FAPs with or without TBHQ (MAPK/ERK activator) treatment. (d, e) The Oil Red O (ORO) staining and statistical analysis for adipogenically differentiated FAPs with different treatments (*n* = 6). *Piezo1 KO* FAPs were induced in adipogenic induction medium for 7 days with or without TBHQ. FAPs from *Piezo1 f/f* mice were also adipogenically differentiated as a control. Scale bar = 100 μm. Absorbance measurement of differentiated *Piezo1 KO* FAPs with or without TBHQ treatment after ORO staining was presented (*n* = 3). (f) The relative protein expression of PPARG, GAPDH and FABP4 in murine *Piezo1 KO* FAPs with or without TBHQ treatment and the *Piezo1 f/f* group (*n* = 3). CTRL, control group. ‘ns’ indicates *p* > 0.05. *p < 0.05, **p < 0.01 and ***p < 0.001.

To investigate the mechanism by which Piezo1 mediates the adipogenesis of FAPs, bulk RNA‐seq of *Piezo1 f/f* (Piezo1^flox/flox^) FAPs and *Piezo1 KO* FAPs was first analysed (Figure 4a). KEGG enrichment analysis of DEGs identified enrichment in MAPK pathways in FAPs after Piezo1 knockout (Figure 4b), which was also enriched in bulk RNA‐seq analysis comparing FAPs with or without RCT (Figure S5a). It has been reported that MAPK/ERK pathways participated in mediating adipogenesis of many cell lineages [25, 26]. These findings were substantiated by Western blotting when comparing the MAPK/ERK signalling between *Piezo1 f/f* FAPs and *Piezo1 KO* FAPs (Figures 4c and S5b), which indicated that decreased expression of PIEZO1 significantly inhibited the activity of MAPK/ERK signalling in FAPs.

To further investigate the function of MAPK/ERK signalling in adipogenesis of FAPs, MAPK/ERK activator TBHQ was applied to activate the MAPK/ERK pathways of *Piezo1 KO* FAPs during adipogenic differentiation. Western blot assays revealed increased phosphorylated ERK1/2 after TBHQ treatment (Figure 4c). The ORO staining assays revealed that there was less lipid in *Piezo1 KO* FAPs after activating the MAPK/ERK pathway by TBHQ (Figure 4d,e). Western blot results also found suppressed expression of adipogenic protein after activating the MAPK/ERK pathway (Figure 4f). However, no difference in adipogenic differentiation capacity was observed between the TBHQ‐treated *Piezo1 KO* group and the *Piezo1 f/f* group, indicating that activating the MAPK/ERK signalling could significantly counteract the effects of *Piezo1 KO* in adipogenesis (Figure 4d–f). Taken together, these studies suggested that Piezo1 regulated the adipogenesis of FAPs through MAPK/ERK pathways. Thus, it could serve as a promising target to reduce fatty infiltration and improve the outcome of RCT.

### 3.4 KLF4 Regulates Piezo1‐Mediated Antiadipogenic Effects in FAPs

**FIGURE 5:**
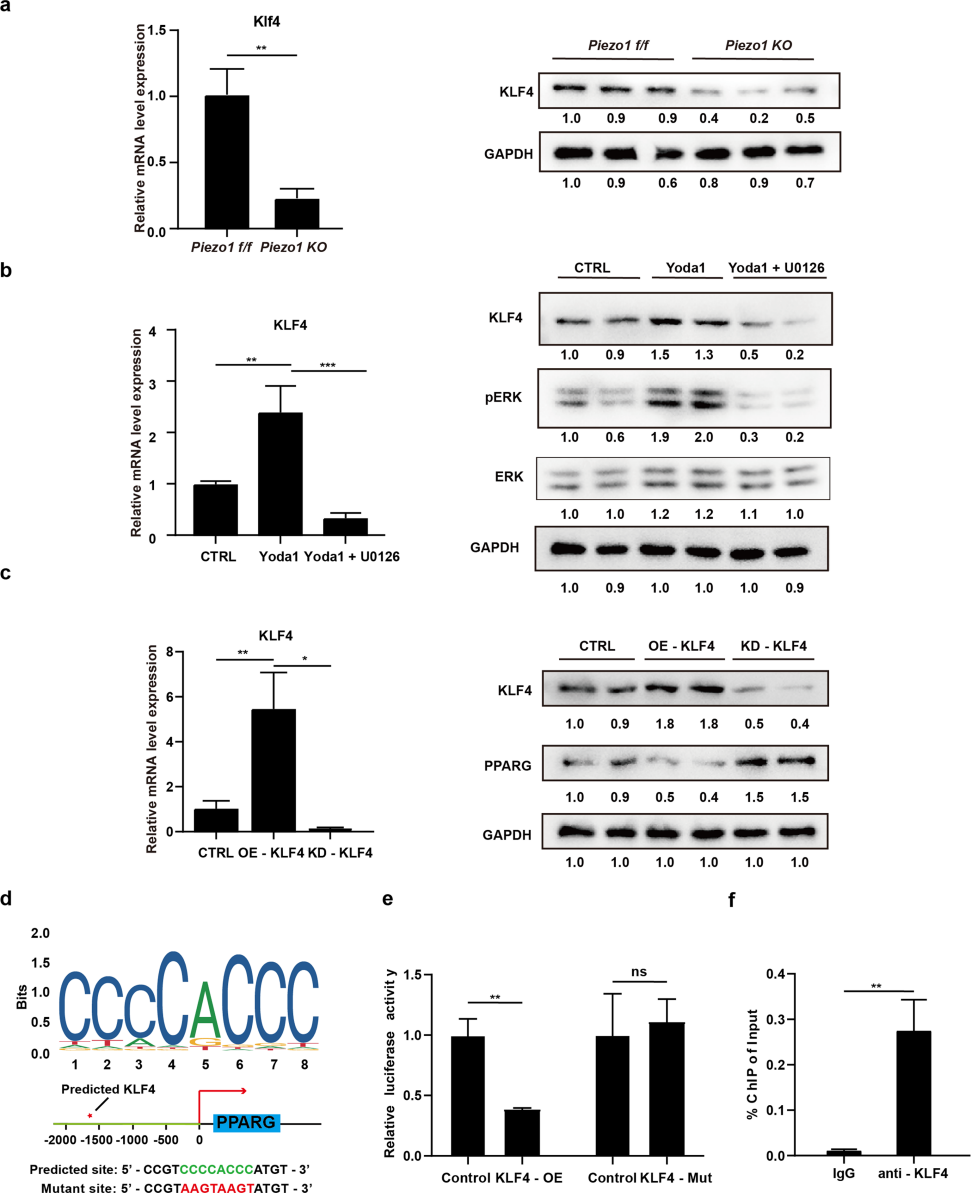
KLF4 regulates Piezo1‐mediated antiadipogenic effects in FAPs. (a) Real‐time qPCR and Western blot analyses of KLF4 expression in FAPs between *Piezo1 KO* and *Piezo1 f/f* groups (*n* = 3). (b) Real‐time qPCR and Western blot analyses for FAPs following Yoda1 treatment or Yoda1 combined with U0126 (MAPK/ERK inhibitor [*n* = 3]) treatment. (c) Real‐time qPCR and Western blot analysis for FAPs following KLF4 overexpression (OE‐KLF4) or KLF4 knockdown (KD‐KLF4) (*n* = 4). (d) The predicted site on the PPARG promoter for KLF4 binding and the diagram of the designed mutant site. (e) Dual‐luciferase reporter assay showing the regulation of KLF4 to PPARG promoter (*n* = 3). (f) Chromatin immunoprecipitation–qPCR analysis for KLF4 binding to the PPARG promoter in FAPs treated with IgG or anti‐KLF4 (*n* = 3). CTRL, control group. ‘ns’ denotes *p* > 0.05. *p < 0.05, **p < 0.01 and ***p < 0.001.

KLF4 is known to mediate stem cell adipogenesis and can be influenced by ERK signalling [27, 28]. Thus, we hypothesized that KLF4 might participate in Piezo1's antiadipogenic effects through ERK signalling. As expected, the bulk RNA‐seq data indicated that KLF4 was significantly decreased after knockout of Piezo1 in FAPs (Figure S6a), which was also verified by results of RT‐qPCR and Western blot (Figure 5a). Consistently, the expression of KLF4 decreased in human FAPs after RCT (Figure S6b,c). Furthermore, activation of PIEZO1 caused by Yoda1 increased the phosphorylation of ERK1/2, accompanied by increased expression of KLF4 (Figure 5b). Conversely, expression of KLF4 was inhibited by MAPK/ERK inhibitor U0126 and even treatment with Yoda1 (Figure 5b). These findings indicated that activation of Piezo1 promoted the phosphorylation of ERK1/2, which further contributed to increased expression of KLF4. To further clarify the relationship between PIEZO1, MAPK/ERK signalling and KLF4, another set of combinatorial small‐molecule treatments was used. Western blot analysis revealed that Piezo1 inhibitor GsMTx4 decreased ERK1/2 phosphorylation and downregulated KLF4 expression, although this effect was offset by the MAPK/ERK activator TBHQ (Figure S6d). These results demonstrate that KLF4 is downstream of the PIEZO1/MAPK/ERK signalling pathway, potentially participating in the regulation of adipogenic differentiation in FAPs.

Then we explored whether KLF4 mediated the adipogenesis of FAPs. Because we found significantly upregulated PPARG expression after KLF4 knockdown and significantly downregulated PPARG expression after KLF4 overexpression (Figure 5c), we then investigated how KLF4 regulated the expression of PPARG. Because KLF4 could act as a transcription factor, the JASPAR database was used, and it indicated potential binding sites of KLF4 on the PPARG promoter (Figure 5d). Then the luciferase reporter assays were carried out using a vector containing firefly luciferase driven by the target gene promoter. Overexpression of KLF4 significantly decreased the luciferase activity (Figure 5e), indicating that KLF4 could suppress the transcriptional activity of PPARG. Furthermore, ChIP–qPCR results revealed that KLF4 could directly bind to the promoter sequence of PPARG (Figure 5f). Taken together, these data indicated that KLF4 could act as a downstream regulator of Piezo1‐mediated antiadipogenic effects in FAPs.

### 3.5 LIPUS Activates PIEZO1 and Inhibits Adipogenesis of FAPs

**FIGURE 6:**
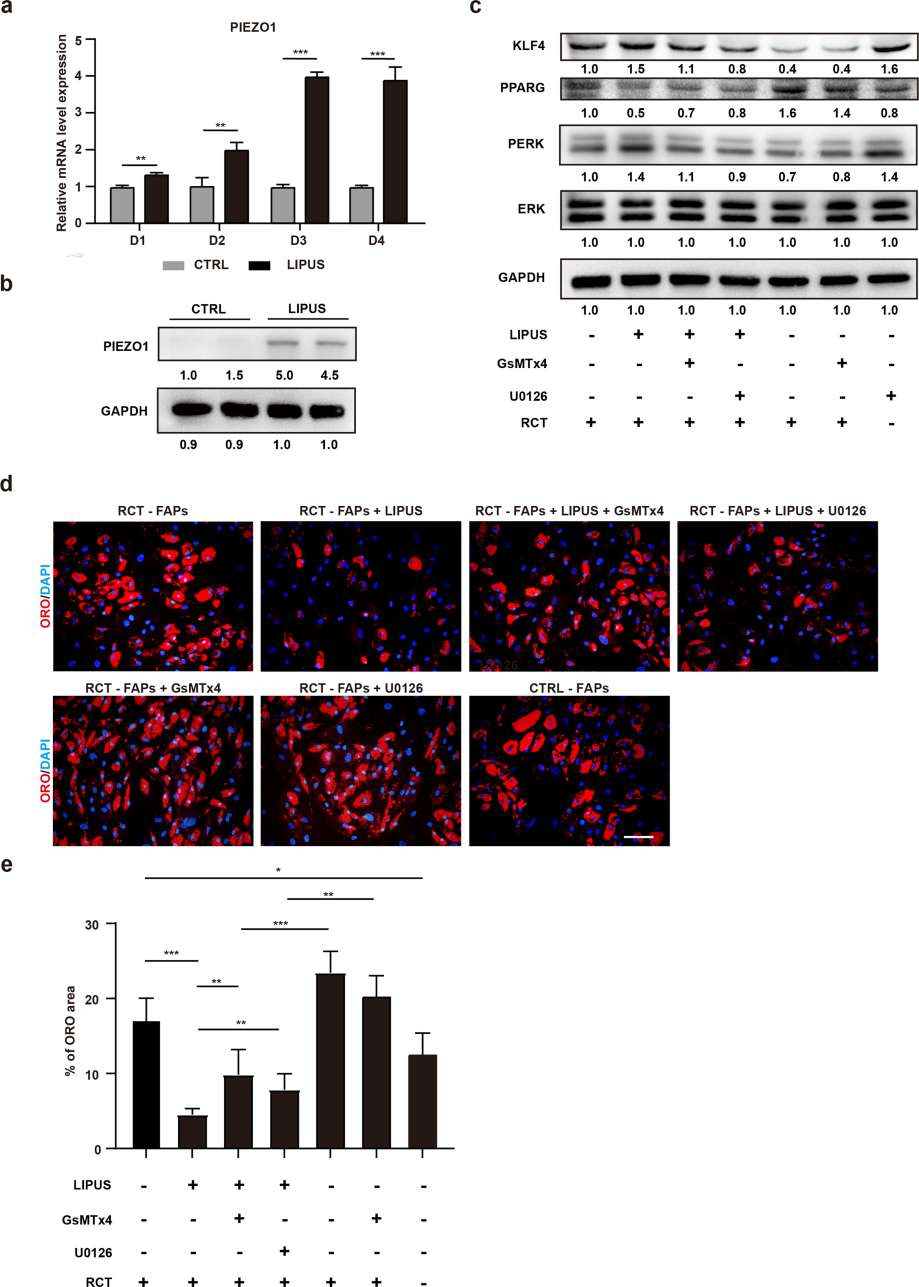
Low‐intensity pulsed ultrasound activates PIEZO1 and inhibits adipogenesis of FAPs. (a) The mRNA expression of PIEZO1 in human FAPs was measured by RT‐qPCR analysis with or without treatment of low‐intensity pulsed ultrasound (LIPUS) for 1, 2, 3 and 4 days (*n* = 3). (b) The protein levels of PIEZO1 and GAPDH in human FAPs with or without LIPUS treatment for 3 days (*n* = 3). (c) The protein levels of KLF4, PPARG, ERK1/2, p‐ERK1/2 and GAPDH in human FAPs after different treatments (*n* = 3). Human RCT‐FAPs were treated with AIM (RCT‐FAPs), AIM + LIPUS (RCT‐FAPs + LIPUS), AIM + LIPUS + GsMTx4 (Piezo1 inhibitor) (RCT‐FAPs + LIPUS + GsMTx4), AIM + LIPUS + U0126 (MAPK/ERK pathway inhibitor) (RCT‐FAPs + LIPUS + U0126), AIM + GsMTx4 (RCT‐FAPs + GsMTx4) and AIM + U0126 (RCT‐FAPs + U0126). (d) Oil Red O (ORO) staining of FAPs after 10 days of adipogenesis with different treatments (*n* = 6). Scale bar = 100 μm. Human RCT‐FAPs were treated with AIM (RCT‐FAPs), AIM + LIPUS (RCT‐FAPs + LIPUS), AIM + LIPUS + GsMTx4 (RCT‐FAPs + LIPUS + GsMTx4), AIM + LIPUS + U0126 (RCT‐FAPs + LIPUS + U0126), AIM + GsMTx4 (RCT‐FAPs + GsMTx4) and AIM + U0126 (RCT‐FAPs + U0126). (e) The statistical analysis for ORO‐positive area of differentiated FAPs. CTRL, control group; CTRL‐FAPs, control group (FAPs isolated from supraspinatus muscle without RCT were treated with AIM). *p < 0.05, **p < 0.01 and ***p < 0.001.

A recent study has reported that LIPUS could activate the Piezo1 and MAPK signalling in the murine preosteoblast cell line MC3T3‐E1 [29]. Thus, the LIPUS was applied in human FAPs to investigate whether it could activate PIEZO1. RT‐qPCR and Western blot results demonstrated that the expression of PIEZO1 was upregulated in human FAPs following LIPUS treatment (Figure 6a,b). To investigate whether the LIPUS plays a significant role in the adipogenic differentiation of FAPs by PIEZO1/MAPK/ERK pathways, the human FAPs were cultured with an adipogenic cocktail with different treatment groups. LIPUS induced the phosphorylation of ERK1/2 and upregulated the protein level of KLF4 (Figure 6c). The activation effects of LIPUS significantly inhibited the adipogenesis of RCT‐FAPs, which was close to the adipogenic differentiation ability of FAPs from healthy control (Figure 6d,e). In contrast, this effect of LIPUS could be attenuated by the application of PIEZO1 inhibitor GsMTx4 and MAPK inhibitor U0126 (Figure 6c–e). These data demonstrated that LIPUS could activate the PIEZO1–ERK–KLF4 axis (Figure 6c–e). However, when the PIEZO1 inhibitor GsMTx4 or MAPK/ERK inhibitor U0126 was used to impair the activation of PIEZO1/MAPK/ERK/KLF4 signalling (Figure 6c), the antiadipogenic effect of LIPUS could be partly reversed (Figure 6d,e). Taken together, these findings indicated that the LIPUS could suppress the adipogenic differentiation of FAPs by activating the PIEZO1–ERK–KLF4 axis.

### 3.6 LIPUS Attenuates the Fatty Infiltration of RCT and Ameliorates Shoulder Functions

**FIGURE 7:**
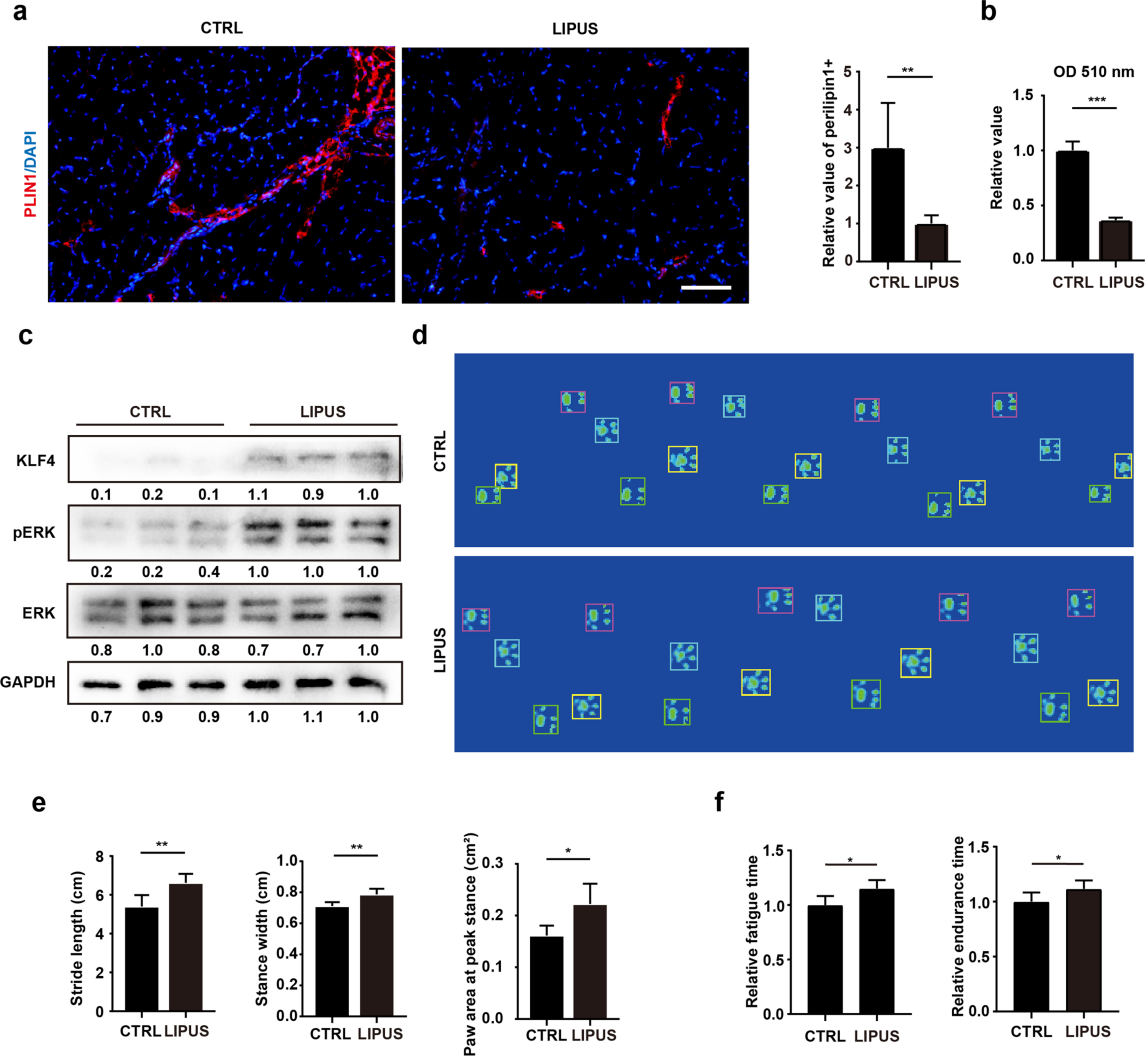
LIPUS attenuates the fatty infiltration of RCT and ameliorates the shoulder functions. (a) The immunofluorescence staining and statistical analysis of Plin1 in supraspinatus muscles after RCT with or without low‐intensity pulsed ultrasound (LIPUS) treatment (*n* = 5). Scale bar = 50 μm. (b) The quantification analysis of triglycerides in supraspinatus muscles from CTRL and LIPUS groups (*n* = 5). (c) The protein levels of KLF4, ERK1/2, p‐ERK1/2 and GAPDH in supraspinatus muscles after RCT with or without LIPUS treatment (*n* = 3). (d, e) The gait analysis for RCT mice with or without LIPUS treatment (*n* = 5). The parameters including stride length, stance width and the paw area at peak stance were recorded and analysed. (f) Fatigue and endurance running time of mice with or without LIPUS treatment (*n* = 5). CTRL, control group. *p < 0.05, **p < 0.01 and ***p < 0.001.

To evaluate whether the antiadipogenic effect of LIPUS on FAPs could alleviate muscular fatty infiltration after RCT in vivo, the RCT mouse models were firstly established. RCT mice were euthanized after 2 months of LIPUS treatment, and the supraspinatus muscle was harvested for subsequent evaluation. Muscular fatty infiltration was firstly measured. The positive area of Plin1 in the supraspinatus muscle after RCT significantly decreased after LIPUS treatment (Figure 7a), indicating a reduced fatty infiltration in RCT. This finding was further supported by triglyceride assays of the supraspinatus muscle (Figure 7b). Immunoblotting assays verified that LIPUS activated the PIEZO1/ERK/KLF4 pathways of FAPs compared to the CTRL group (Figure 7c). Thus, these results demonstrated that LIPUS could suppress excessive fatty infiltration after RCT.

Because muscular fatty infiltration significantly affected shoulder function [4], gait analysis and treadmill tests were performed to evaluate the therapeutic effects of LIPUS for RCT mice. In gait analysis, the stride length signifies the degree of shoulder abduction in mice, whereas the stance width illustrates the limb's load‐bearing capacity. Additionally, the paw area at peak stance serves as an indicator of chronic pain. There were significant improvements in stride length, stance width and paw area at peak stance after LIPUS treatment (Figure 7d,e). In addition, treadmill tests showed that the endurance and exhaustion time were also increased after LIPUS treatments, indicating improved shoulder functions caused by LIPUS (Figure 7f).

Altogether, these data revealed that LIPUS could abate muscular fatty infiltration and ameliorate shoulder function in RCT models.

## 4 Discussion

The current study demonstrated that the expression of PIEZO1 was downregulated in both human and murine FAPs after RCT, which contributed to increased conversion of FAPs to adipocytes by inhibiting ERK/KLF4 pathways. LIPUS could abate the excessive adipogenesis of FAPs and alleviate muscular fatty infiltration after RCT by upregulating the expression of PIEZO1, ultimately leading to enhanced shoulder functions. LIPUS could be a promising non‐invasive intervention to ameliorate the muscular fatty infiltration of RCT.

RCT is a common ailment in orthopaedic surgery, characterized by chronic shoulder pain and disability [8]. Surgical treatment is a primary therapeutic modality for RCT. However, the retearing rate following RCT repair still remains notably high, even with advancements in operative techniques [30]. Irreversible muscular fatty infiltration after RCT is recognized as a significant factor contributing to poor surgical outcomes [23]. Nevertheless, addressing muscular fatty infiltration in clinical practices presents challenges due to the lack of understanding of in‐depth mechanisms underlying fatty degeneration. Therefore, it is essential to explore the mechanism of muscle degeneration after RCT and propose auxiliary treatments to reduce muscular fatty infiltration after RCT, which motivated the conduct of this study.

FAPs are the primary source of muscular fatty infiltration following RCT. Studies have indicated that the proliferative and differentiative capacities of FAPs are closely associated with the sizes of tendon tears [31]. In addition, FAPs exhibit a heightened adipogenic potential after RCT compared to Achilles tendon tears [15]. Inhibition of adipogenic differentiation of FAPs using agents such as WNT agonist (BML‐284) [15], retinoic receptor agonist (adapalene) [32] and PDGFRα pathway inhibitor (imatinib) [33] has shown potential to alleviate fatty degeneration in RCT. These findings highlight FAPs as a promising target for investigating the underlying mechanisms of fatty infiltration after RCT. FAPs presented significantly different functions under different diseases. A previous study has indicated that FAPs promoted muscle atrophy and fibrosis of skeletal muscle in the state of nerve injury [34]. Additionally, high‐Sca‐1 FAPs collected from Duchenne muscular dystrophy mice showed higher proliferative and adipogenic potentials, whereas low‐Sca‐1 FAPs presented considerable fibrogenic differentiation potential [6]. Furthermore, FAPs obtained from elderly individuals have been found to exhibit reduced WISP1 secretion, thus impairing their potential to rejuvenate myogenesis [35]. In the current study, increased adipogenesis of FAPs was identified in both humans and mice after RCT. This finding robustly supports the hypothesis that increased transformation of FAPs into adipocytes contributes to the development of muscular fatty infiltration after RCT, emphasizing the distinct characteristics of FAPs in different diseases.

In this study, we found that PIEZO1 was downregulated in FAPs isolated from RCT, thereby fostering enhanced adipogenic differentiation potentials of FAPs in vivo and in vitro. Because RCT inevitably destroys the biomechanical microenvironment of rotator cuff muscles, it is reasonable that RCT consequently induces an altered biological effect through mechanosensory protein PIEZO1 [17]. PIEZO1 is a well‐known mechanosensory protein integral in transducing mechanical signals and mediating physiological activities [36]. It is worth noting that activation of PIEZO1 could significantly reduce the adipogenic potential of preadipocytes derived from perivascular adipose tissue [36]. Additionally, PIEZO1 promoted osteogenic differentiation while mitigating the adipogenesis of bone mesenchymal stem cells [24]. Thus, these studies indicated that PIEZO1 could mediate adipogenesis of different cells. However, it remains to be explored whether PIEZO1 exerts a similar antiadipogenic effect in FAPs before the current investigation. To the best of our knowledge, it is the first study that clarifies the direct regulatory role of PIEZO1 on adipogenesis of FAPs, which also sheds light on the mechanism of excessive muscular fatty infiltration after RCT.

KLF4 is a well‐established transcription factor that plays a crucial role in regulating stem cell function, particularly in maintaining pluripotency and self‐renewal [37]. It is one of the key factors in the Yamanaka cocktail, which reprograms somatic cells into induced pluripotent stem cells. In addition to its role in pluripotency, KLF4 has been shown to influence the balance between stem cell proliferation and differentiation, acting as a switch that either promotes or inhibits differentiation depending on the cellular context. Its involvement in various signalling pathways, including Wnt, Notch and MAPK, underscores its pivotal role in stem cell fate determination. In our study, KLF4 was found to mediate the antiadipogenic effects of Piezo1 through ERK1/2, further demonstrating its functional versatility in regulating mesenchymal stem cell differentiation. By inhibiting adipogenesis, KLF4 may help maintain the stemness of FAPs, thereby preserving their regenerative potential in various tissues.

The suppressed expression of PIEZO1 in FAPs is more likely to be attributed to reduced load transmission of rotator cuff muscles after RCT, indicating the application potential of mechanical stimuli to counteract FAP adipogenesis and muscular fatty degeneration. Thus, LIPUS, which could provide mechanical stimuli, was investigated to abate excessive muscular fatty infiltration in the current study. LIPUS is widely used to treat musculoskeletal diseases, particularly in promoting bone healing [38], bone–tendon interface healing [39] and muscle degeneration [40]. However, its application in RCT is rare. Here, we demonstrated that LIPUS could alleviate the adipogenic differentiation of FAPs by activating the PIEZO1–ERK–KLF4 axis and thus attenuating the muscular fatty infiltration and enhancing shoulder function after RCT. Because LIPUS is a painless and non‐invasive intervention, it showed promising clinical application prospects in the future. However, further studies should be performed to explore the frequency, safety and feasibility of LIPUS for delaying or alleviating muscular fatty infiltration in RCT.

In conclusion, the current study demonstrated that the expression of PIEZO1 was downregulated in FAPs after RCT, which contributed to increased conversion of FAPs to adipocytes by inhibiting ERK/KLF4 pathways. LIPUS could abate the excessive adipogenesis of FAPs and alleviate muscular fatty infiltration after RCT by upregulating expression of PIEZO1, ultimately leading to enhanced shoulder functions.

## Conflicts of Interest

The authors declare no conflicts of interest.

## Supporting information


**Figure S1.** The isolation and identification of FAPs from human and mouse muscles. (a) The isolation strategies of human FAPs by FACS. (b) Mouse FAPs isolation strategies. (c) and (d) The immunofluorescence staining and statistical analysis of PDGFRα for isolated human and mouse FAPs (*n* = 5). Scale Bar = 100 μm.
**Figure S2.** There was increased adipogenesis ability of murine FAPs after RCT. (a) and (b) The oil red O (ORO) staining and quantitative analysis of murine FAPs isolated from CTRL and RCT groups after 7‐day adipogenic induction (*n* = 5). Scale bar = 100 μm. (c) Absorbance measurement of differentiated FAPs from CTRL and RCT groups after ORO staining (*n* = 3). (d) Relative gene and protein expression of PPARG, FABP4, and GAPDH of FAPs from CTRL and RCT groups after 7‐day adipogenesis (*n* = 3). (e) Protein expression of FABP4, PPARG, and GAPDH in FAPs isolated from CTRL and RCT mice (*n* = 3). The * indicates P < 0.05, ** indicates P < 0.01, and *** indicates P < 0.001.
**Figure S3.** The PIEZO1 is required for the adipogenesis of FAPs. (a)‐(c) Oil Red O (ORO) staining and quantitative analysis of FAPs induced with adipogenic induction medium (AIM) for 10 days (*n* = 6). Freshly isolated healthy FAPs with PIEZO1 overexpression (OE) or knockdown (KD) were cultured in AIM for 10 days prior to analysis. Scale bar = 100 μm. (d) Relative protein expression of PIEZO1, PPARγ, GAPDH, and FABP4 in FAPs treated with PIEZO1 knockdown (KD) or overexpression (OE) (*n* = 3). The ns indicates P > 0.05, * indicates P < 0.05, ** indicates P < 0.01, and *** indicates P < 0.001.
**Figure S4.** FAPs obtained higher adipogenic differentiation potential after Piezo1 ablation. (a) and (b) Immunofluorescence staining of Pparg and PDGFRα in supraspinatus muscles from *Piezo1 f/f* and *Piezo1KO* mice (*n* = 5). Scale bar = 50 μm. Arrows indicate Pparg positive FAPs cells. The * indicates P < 0.05 and *** indicates P < 0.001.
**Figure S5.** RNA‐seq analysis revealed decreased activity in MAPK/ERK pathways for FAPs after RCT. (a) Bubble chart of KEGG analysis for down‐regulated genes of murine FAPs with RCT when compared with those in CTRL model. (b) The protein expression of ERK1/2, p‐ERK1/2, and GAPDH in FAPs isolated from CTRL and RCT groups (*n* = 3). The * indicates P < 0.05, ** indicates P < 0.01, and *** indicates P < 0.001.
**Figure S6.** KLF4 is downstream of PIEZO1/ERK pathway in FAPs. (a) The heatmap of differentially expressed genes in FAPs obtained from *Piezo1 f/f* and *Piezo1 KO* mice. (b) and (c) Relative gene and protein expression of KLF4 in human FAPs from supraspinatus muscle with or without RCT (*n* = 3). (d) Relative protein expression of KLF4, ERK, phospho‐ERK (p‐ERK), and GAPDH in human FAPs treated with various small molecule compounds (*n* = 3). GsMTx4, PIEZO1 inhibitor; TBHQ, ERK MAPK activator.
**Table S1.** The clinical information of human sample.

## Data Availability

The datasets used in this study can be obtained from the corresponding author upon reasonable request. The single‐cell RNA‐seq data and bulk RNA‐seq data have been uploaded to the Sequence Read Archive database. The accession numbers are PRJNA1247938 and PRJNA1250201.
