# Supplementary material for: Unravelling the Role of PIEZO1 in Adipogenesis of Fibrogenic/Adipogenic Progenitors for Muscle Fat Infiltration After Rotator Cuff Tear
Source: J Cachexia Sarcopenia Muscle. 2025 Jul 29;16(4):e70004. doi: 10.1002/jcsm.70004 (PMC12304729; doi:10.1002/jcsm.70004)
Supplement: Supplementary file 1 — Figure S1. The isolation and identification of FAPs from human and mouse muscles. (a) The isolation strategies of human FAPs by FACS. (b) Mouse FAPs isolation strategies. (c) and (d) The immunofluorescence staining and statistical analysis of PDGFRα for isolated human and mouse FAPs (n = 5). Scale Bar = 100 μm. Figure S2. There was increased adipogenesis ability of murine FAPs after RCT. (a) and (b) The oil red O (ORO) staining and quantitative analysis of murine FAPs isolated from CTRL and RCT groups after 7‐day adipogenic induction (n = 5). Scale bar = 100 μm. (c) Absorbance measurement of differentiated FAPs from CTRL and RCT groups after ORO staining (n = 3). (d) Relative gene and protein expression of PPARG, FABP4, and GAPDH of FAPs from CTRL and RCT groups after 7‐day adipogenesis (n = 3). (e) Protein expression of FABP4, PPARG, and GAPDH in FAPs isolated from CTRL and RCT mice (n = 3). The * indicates P < 0.05, ** indicates P < 0.01, and *** indicates P < 0.001. Figure S3. The PIEZO1 is required for the adipogenesis of FAPs. (a)‐(c) Oil Red O (ORO) staining and quantitative analysis of FAPs induced with adipogenic induction medium (AIM) for 10 days (n = 6). Freshly isolated healthy FAPs with PIEZO1 overexpression (OE) or knockdown (KD) were cultured in AIM for 10 days prior to analysis. Scale bar = 100 μm. (d) Relative protein expression of PIEZO1, PPARγ, GAPDH, and FABP4 in FAPs treated with PIEZO1 knockdown (KD) or overexpression (OE) (n = 3). The ns indicates P > 0.05, * indicates P < 0.05, ** indicates P < 0.01, and *** indicates P < 0.001. Figure S4. FAPs obtained higher adipogenic differentiation potential after Piezo1 ablation. (a) and (b) Immunofluorescence staining of Pparg and PDGFRα in supraspinatus muscles from Piezo1 f/f and Piezo1KO mice (n = 5). Scale bar = 50 μm. Arrows indicate Pparg positive FAPs cells. The * indicates P < 0.05 and *** indicates P < 0.001. Figure S5. RNA‐seq analysis revealed decreased activity in MAPK/ERK pathways for FAPs [file JCSM-16-e70004-s001.docx]

**
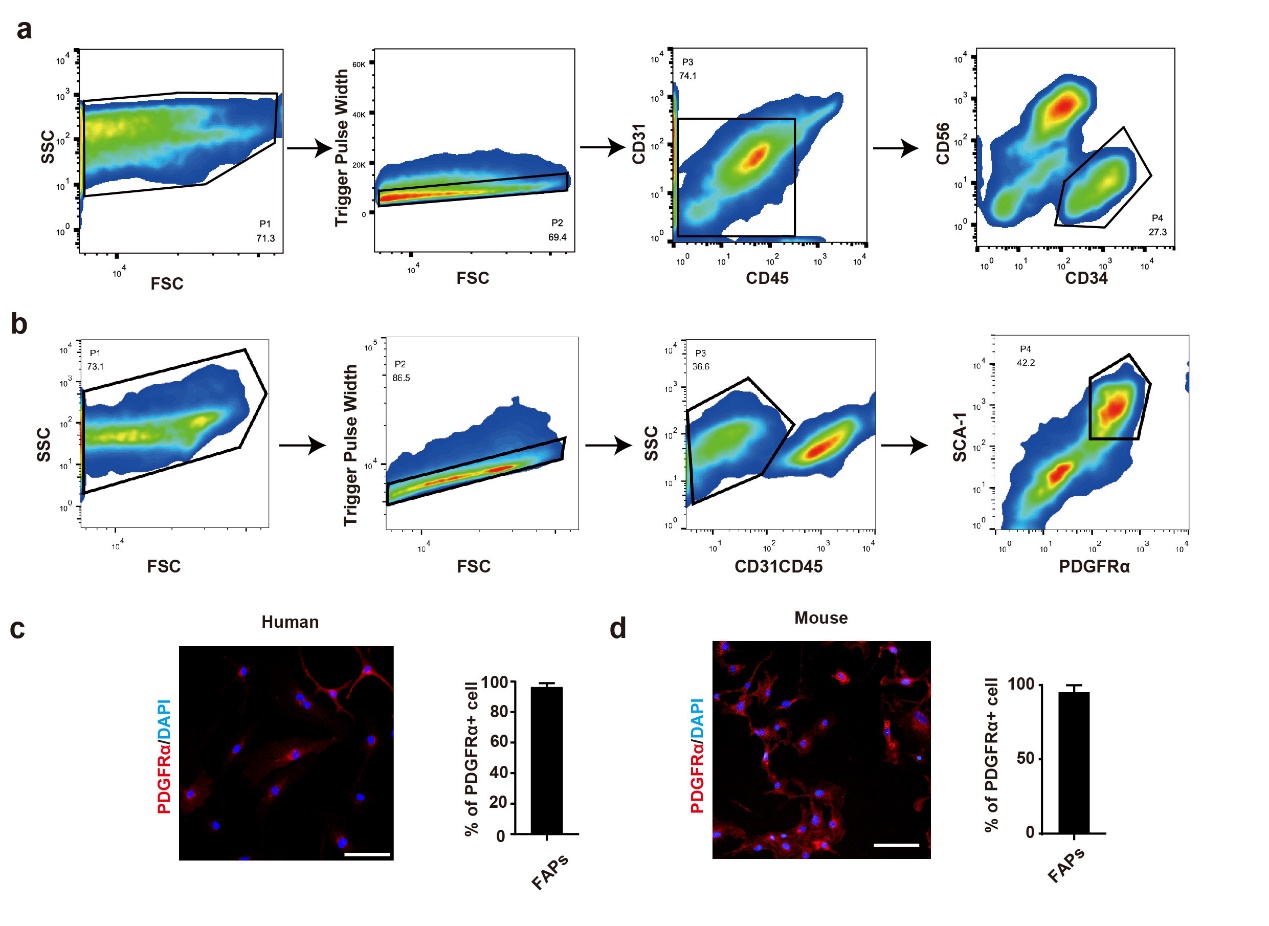
sFigure 1 The isolation and identification of FAPs from human and mouse muscles.**

(a) The isolation strategies of human FAPs by FACS.

(b) Mouse FAPs isolation strategies.

(c) and (d) The immunofluorescence staining and statistical analysis of PDGFRα for isolated human and mouse FAPs (n=5). Scale Bar = 100 µm


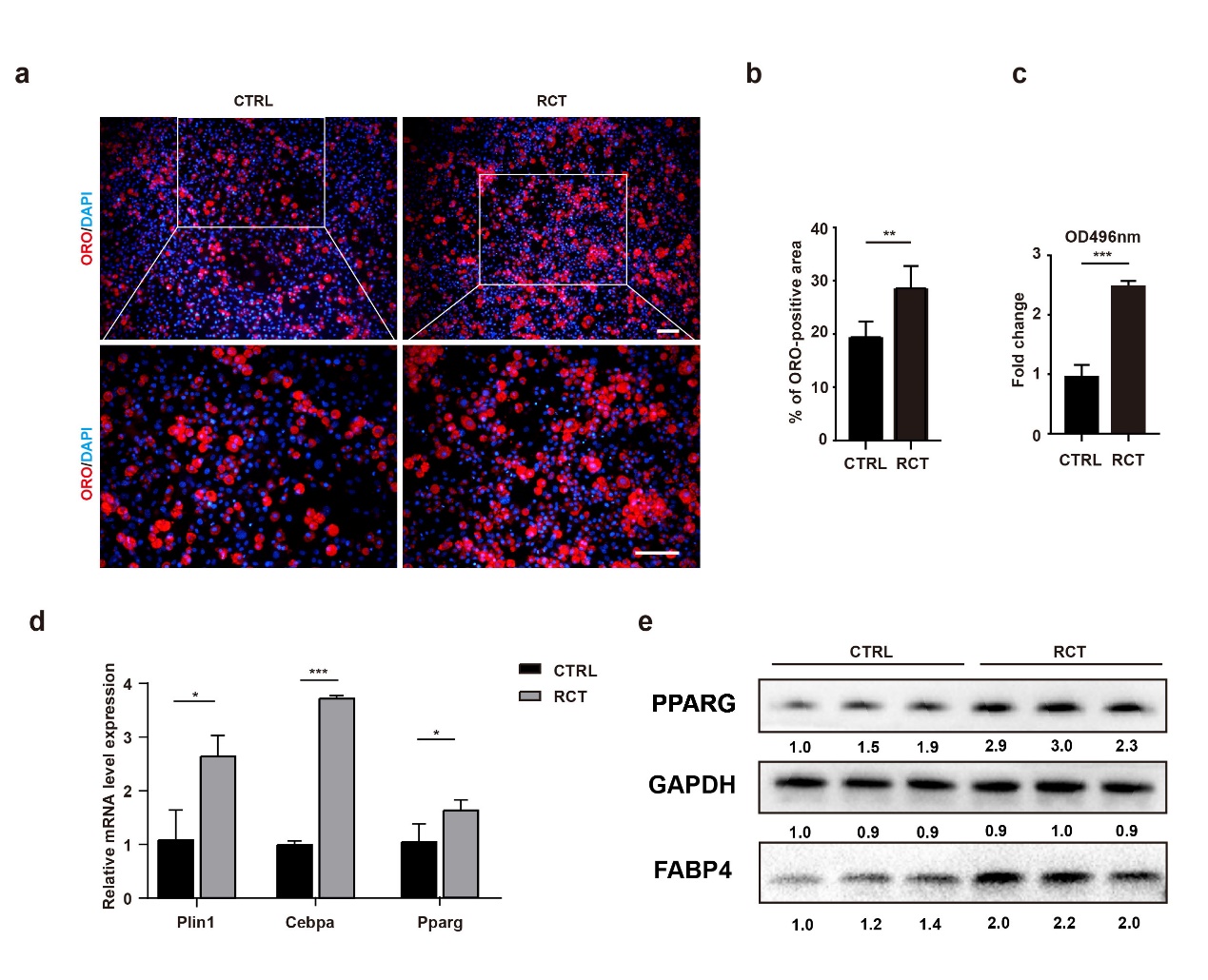


**sFigure 2 There was increased adipogenesis ability of murine FAPs after RCT.**

(a) and (b) The oil red O (ORO) staining and quantitative analysis of murine FAPs isolated from CTRL and RCT groups after 7-day adipogenic induction (n=5). Scale bar = 100 µm.

(c) Absorbance measurement of differentiated FAPs from CTRL and RCT groups after ORO staining (n=3).

(d) Relative gene and protein expression of PPARG, FABP4, and GAPDH of FAPs from CTRL and RCT groups after 7-day adipogenesis (n=3).

(e) Protein expression of FABP4, PPARG, and GAPDH in FAPs isolated from CTRL and RCT mice (n=3).

The * indicates P＜0.05, ** indicates P＜0.01, and *** indicates P＜0.001.


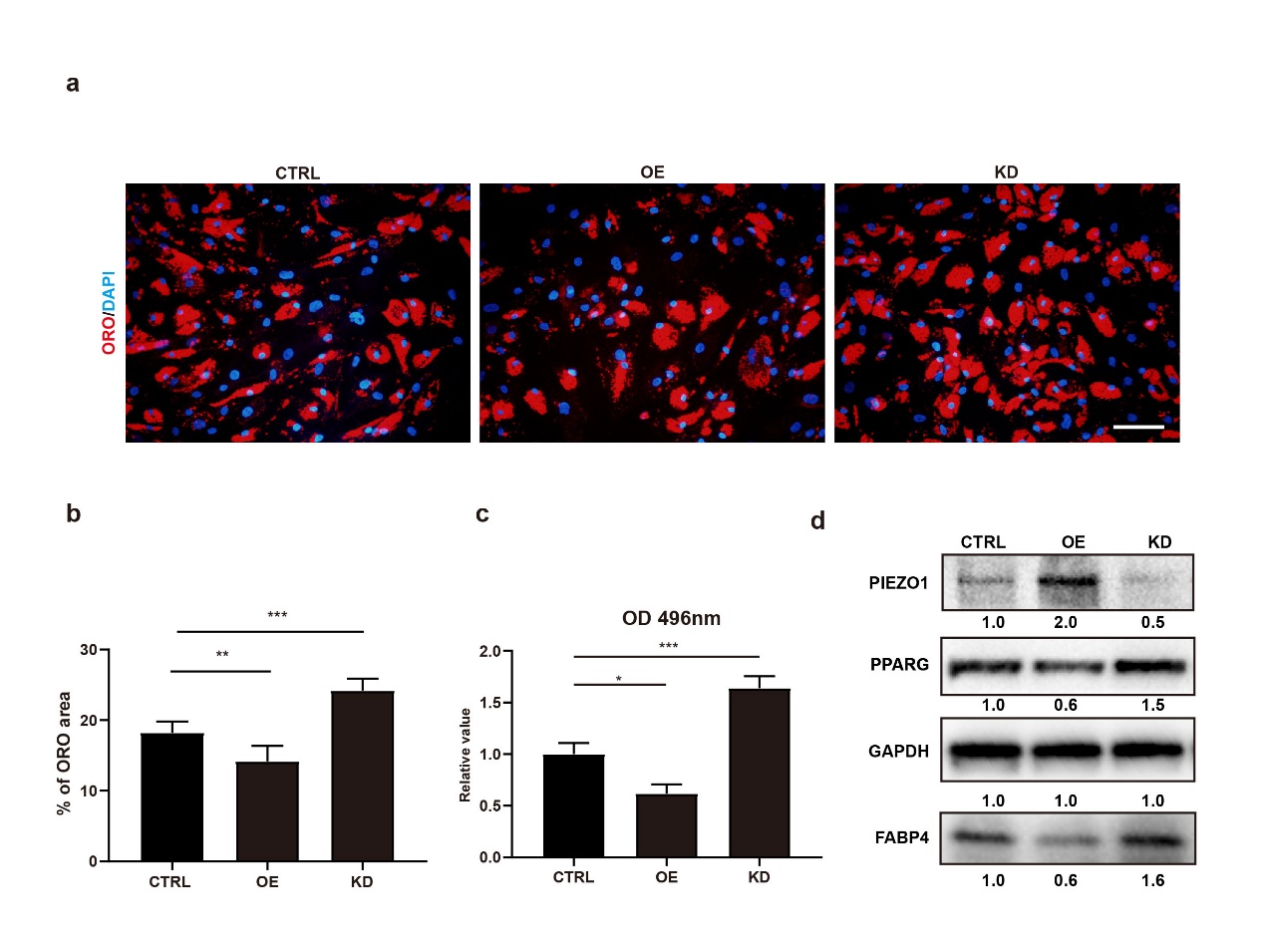


**sFigure 3 The PIEZO1 is required for the adipogenesis of FAPs.**

(a)-(c) Oil Red O (ORO) staining and quantitative analysis of FAPs induced with adipogenic induction medium (AIM) for 10 days (n=6). Freshly isolated healthy FAPs with PIEZO1 overexpression (OE) or knockdown (KD) were cultured in AIM for 10 days prior to analysis. Scale bar = 100 µm.

(d) Relative protein expression of PIEZO1, PPARγ, GAPDH, and FABP4 in FAPs treated with PIEZO1 knockdown (KD) or overexpression (OE) (n=3).

**
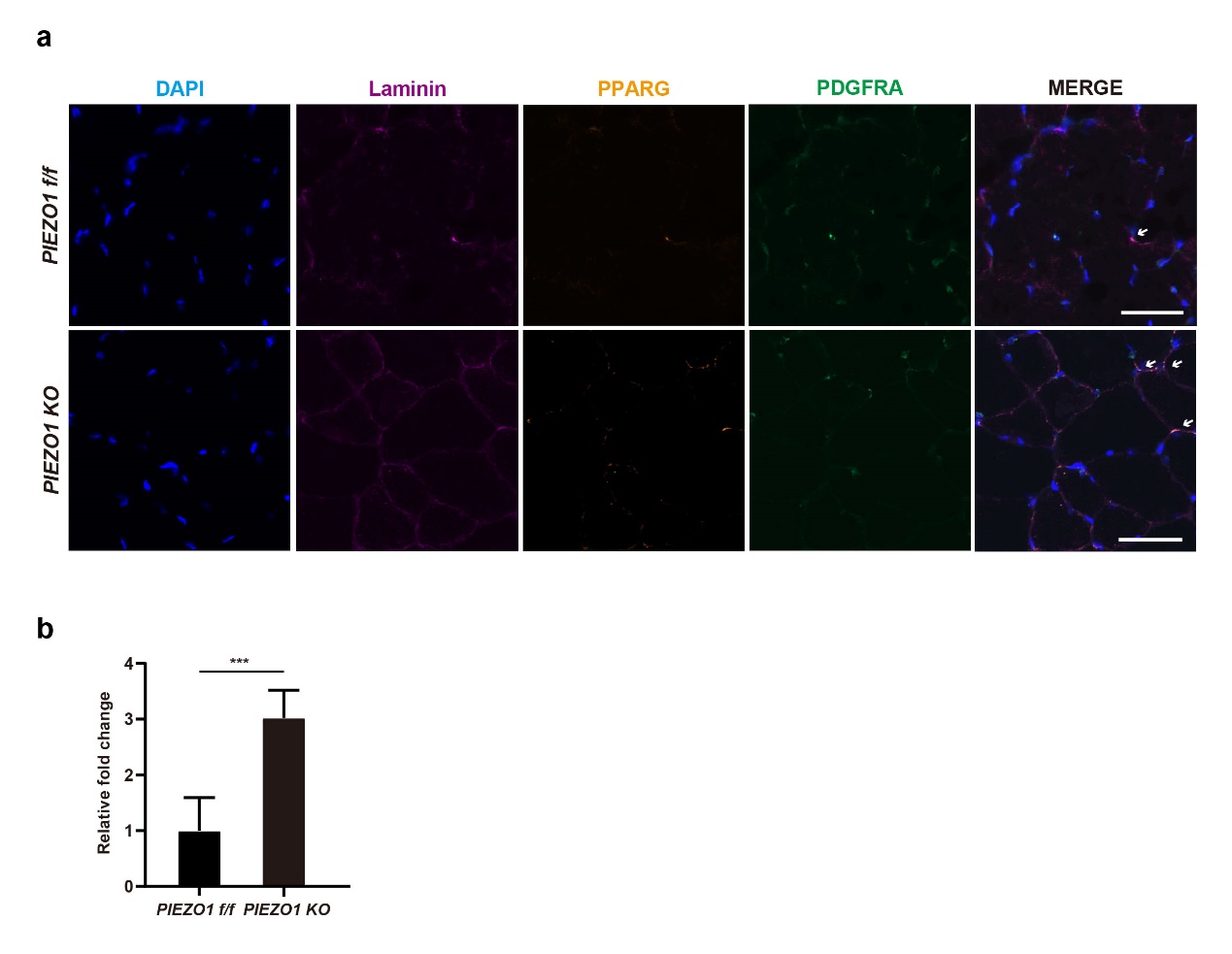
**The ns indicates P＞0.05, * indicates P＜0.05, ** indicates P＜0.01, and *** indicates P＜0.001.

**Figure 4 FAPs obtained higher adipogenic differentiation potential after Piezo1 ablation.**

(a) and (b) Immunofluorescence staining of Pparg and PDGFRα in supraspinatus muscles from *Piezo1 f/f* and *Piezo1KO* mice (n=5). Scale bar = 50 µm. Arrows indicate Pparg positive FAPs cells.

The * indicates P＜0.05 and *** indicates P＜0.001.


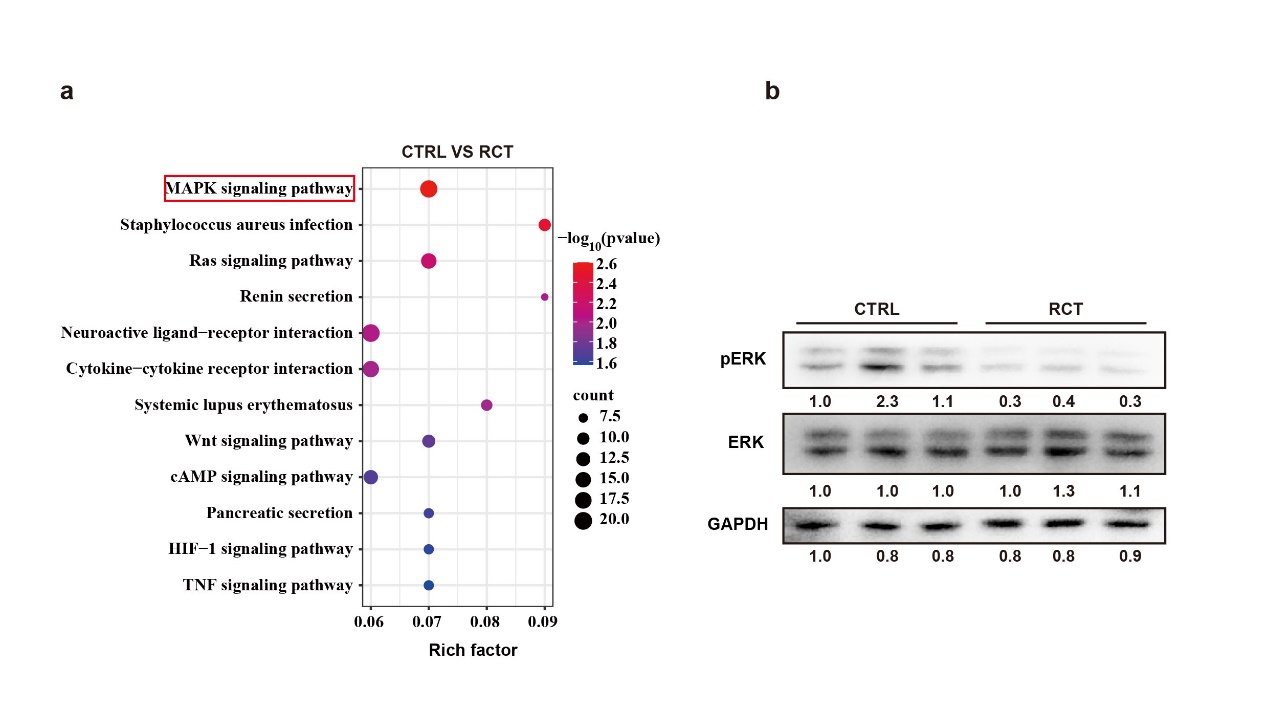


**sFigure 5. RNA-seq analysis revealed decreased activity in MAPK/ERK pathways for FAPs after RCT**

(a) Bubble chart of KEGG analysis for down-regulated genes of murine FAPs with RCT when compared with those in CTRL model.

(b) The protein expression of ERK1/2, p-ERK1/2, and GAPDH in FAPs isolated from CTRL and RCT groups (n=3).

The * indicates P＜0.05, ** indicates P＜0.01, and *** indicates P＜0.001.

**
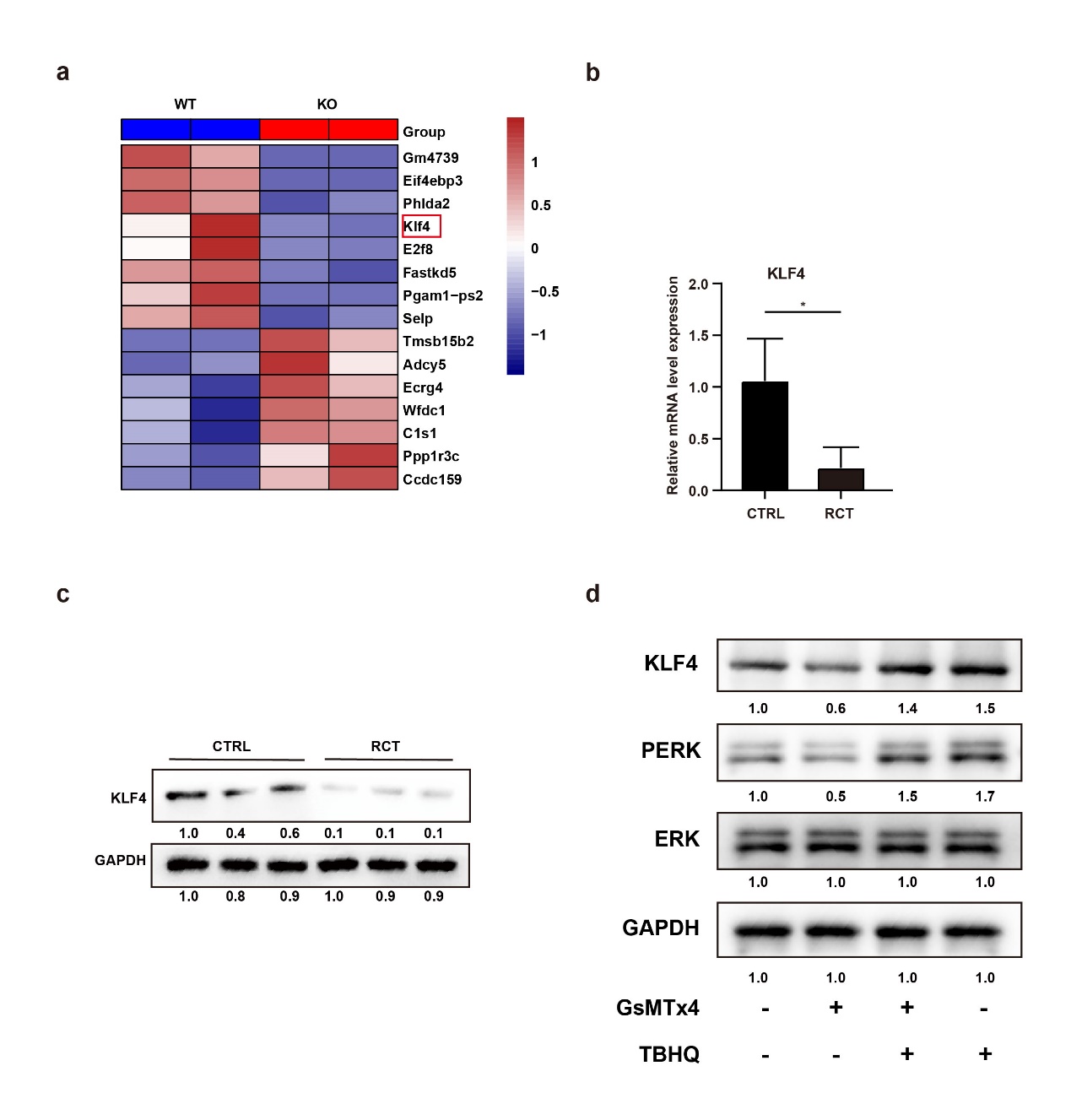
**

**sFigure 6 KLF4 is downstream of PIEZO1/ERK pathway in FAPs.**

(a) The heatmap of differentially expressed genes in FAPs obtained from *Piezo1 f/f* and *Piezo1 KO* mice.

(b) and (c) Relative gene and protein expression of KLF4 in human FAPs from supraspinatus muscle with or without RCT (n=3).

(d) Relative protein expression of KLF4, ERK, phospho-ERK (p-ERK), and GAPDH in human FAPs treated with various small molecule compounds (n=3). GsMTx4, PIEZO1 inhibitor; TBHQ, ERK MAPK activator.

**Supplemental Table**

Table1. The clinical information of human sample

| **Number** | **Age** | **Sex** | **Ethnicity** | Group |
| --- | --- | --- | --- | --- |
| 1 | 56 | Male | Han Chinese | Control |
| 2 | 61 | Female | Han Chinese | Control |
| 3 | 64 | Female | Han Chinese | Rotator cuff tear |
| 4 | 55 | Male | Han Chinese | Rotator cuff tear |
